# Autoencoder-Based Detection of Nanoplastics in Biological Matrices via Infrared Hyperspectral Imaging

**DOI:** 10.1021/acsmeasuresciau.6c00067

**Published:** 2026-07-18

**Authors:** Daniel Prezgot, Sadman Sakib, Maohui Chen, Adrian F. Pegoraro, David Corriveau, Shan Zou

**Affiliations:** Nanoscale Measurement, Metrology Research Centre, 6356 National Research Council Canada, 100 Sussex Drive, Ottawa, ON K1A0R6, Canada

**Keywords:** nanoparticles, nanoplastics, anomaly mapping, spectral unmixing, laser-direct infrared (LDIR) microscopy, biological matrix

## Abstract

Mid-infrared hyperspectral imaging is an emerging tool for the qualitative and quantitative analysis of micro- and nanoplastics (MNPs) and for the characterization of biological tissues and complex matrices. Detecting MNPs in biological materials is of particular interest for assessing toxicological and ecological impacts; however, significant spectral overlap between polymer vibrational bands and those of proteins, lipids, and other biological components complicates identification at low MNP levels. In this work, an autoencoder-based anomaly detection approach is employed to learn the spectral characteristics of biological matrix signals from infrared spectra acquired with quantum cascade laser infrared (QCL-IR) microscopy. Residual-based anomaly mapping preserves chemically meaningful spectral features, enabling heatmap visualization of nanoscale plastic (NP) accumulation in two- and three-dimensional cell culture models. Fully connected (FC), convolutional neural network (CNN) and hybrid (CNN-FC) autoencoder architectures were evaluated, with FC and CNN-FC models providing a balance between accurate biological matrix reconstruction and preservation of NP spectral signatures. The method enabled detection of ∼50 nm plastic particles when present as localized accumulations corresponding to approximately 1% (m/m) of the dried biological material, equivalent to surface density on the order of 10^3^ particles/μm^2^. These results demonstrate that residual anomaly detection can extend hyperspectral imaging to visualize nanoscale plastic accumulations in complex biological media at concentrations approaching the instrumental detection limit.

## Graphical abstract

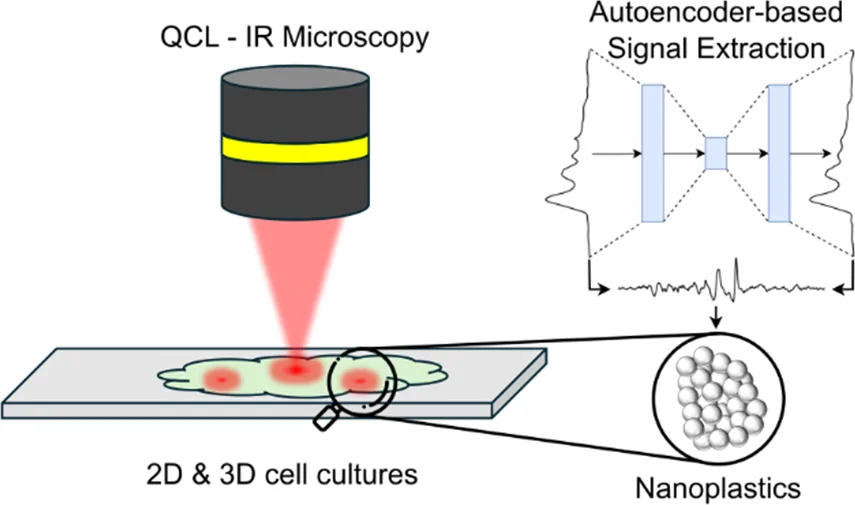

## Introduction

Micro- and nanoplastics (MNPs) are ubiquitous environmental contaminants of increasing concern, as global plastic production and utilization show no sign of slowing.^1–3^ The degradation and fragmentation of environmental plastics produce secondary micro (<5 mm), submicron (<1 μm), and nanoscale (<100 nm) plastics that infiltrate drinking water and food, leading to their uptake by organisms and humans.^4,5^ This raises concerns about the potential impact on ecosystems and human health.^5–9^ The ability of submicron and nanoscale plastics (NPs) to cross biological barriers and accumulate in tissues remains under investigation.^10–13^ The detection of MNP uptake and distribution within organisms is thus an important part of understanding their potential toxicological effects and long-term impacts. To track plastic accumulation in tissues, various microscopy techniques have been employed, including fluorescence microscopy,^14^ electron microscopy,^15^ atomic force microscopy (AFM),^16^ near-IR microscopy,^17^ and hyperspectral imaging.^18^ Among the most powerful microscopy techniques for this purpose are vibrational spectroscopy-based techniques such as Raman,^19^ stimulated Raman,^15^ and mid-infrared (MIR)^20^ microscopy, all of which are capable of providing label-free spatially resolved chemical information in the form of a hyperspectral image. MIR spectroscopy is relatively robust against background species and its use has been driven by recent advances in instrumentation, such as optical-photothermal infrared (O-PTIR) microscopy,^21–23^ and quantum cascade laser infrared (QCL-IR) or laser-direct infrared (LDIR) microscopy.^20,24^

MIR spectroscopy has been used for a variety of cytological and histological applications,^25–28^ and can be applied to detect MNPs in biological media. Detection of submicron and nanoscale plastics is challenging because the diffraction-limited spatial resolution of MIR imaging (∼10–20 μm) exceeds the size of these particles.^29^ Coupling IR with other detection modalities, such as O-PTIR and AFM-IR,^22^ can partially overcome this. MNPs tend to accumulate when internalized in tissues and cells.^30,31^ While isolated single nanoparticles are far below the diffraction limit of conventional MIR imaging, their accumulation into local domains produces optically resolvable signals; thus, detection depends primarily on spectral sensitivity rather than spatial resolution. Their polymer-specific absorbance can also be weak relative to the strong broadband infrared absorption of a surrounding biological matrix. Cellular and biological materials contain abundant lipids, proteins, nucleic acids, and carbohydrates that generate broad overlapping vibrational bands, many of which coincide with characteristic polymer resonances. As a result, low-concentration plastic signatures may be masked by the background absorption of the biological matrix. Existing label-free approaches often lack the sensitivity to reliably detect such weak polymer signatures without extensive preprocessing or threshold-based spectral analysis, highlighting the need for advanced methods capable of measuring low-abundance signals in complex biological matrices. Notably, machine-learning–assisted approaches have been applied to MIR spectroscopy, including TGA-FTIR^32,33^ and MIR microscopy^34–36^ to improve identification and quantification of microplastics, demonstrating the potential of computational techniques to enhance analytical sensitivity and throughput.

Deep learning techniques offer an avenue for enhancing the analysis of MNPs in complex matrices^37^ and have been applied to a number of problems in vibrational spectroscopy, such as background correction,^38–40^ classification,^36,41,42^ and spectral unmixing.^43^ Anomaly detection, is a method for identifying outliers in a sample population. In hyperspectral imaging, it can be applied to detect spectral signatures that deviate from established normal spectra or to identify anomalous pixels within a hyperspectral image.^44^ Anomaly detection has been applied to a number of fields which utilize hyperspectral imaging, including remote sensing,^45,46^ mineralogy,^47^ and biomedical imaging.^48^ This technique can be performed using deep learning techniques via a reconstruction-based approach with autoencoders, in which a model learns a lower dimensional representation of the data (or encodes the data) and then attempts to reconstruct (decode) it back to the original. Models trained on either control or unsupervised data reveal anomalies when reconstruction yields errors that deviate from the original spectrum data. The use of residual spectral reconstruction error, the difference between the original and reconstructed spectra, can identify anomalies while preserving a chemically meaningful spectral structure that can be used to identify the chemical species in the anomalous data.

In this work, residual anomaly detection was applied to visualize the uptake of polypropylene and polystyrene nanoscale plastics across a range of 2D monolayer cultures and 3D organoid models. Rather than employing anomaly detection as a purely unsupervised statistical outlier approach, the framework incorporates prior knowledge of characteristic polymer vibrational bands to guide the interpretation of anomalous spectral features. Residual anomaly detection is implemented by training neural network autoencoders on control data from cells that have not been exposed to NPs. Since the models are trained on data that do not contain plastic, absorption bands from plastic remain in the residual spectra. Importantly, the residual spectra are not reduced to scalar scores but are interrogated at known polymer-specific absorption bands, allowing chemically informed mapping of NP signatures. This chemically guided residual analysis not only identifies anomalies but preserves the polymer-specific spectral structure, enabling heatmap visualization of nanoscale plastic accumulation at subcellular resolution with improved specificity. Because the spectral signatures of plastics are known, creating a heatmap based on selected absorption peaks further improves specificity. This strategy enables high-specificity mapping of nanoscale plastics in biological samples at near-instrumental-detection thresholds.

## Methods

### Materials

Nanoscale plastic polypropylene (NPPP-1)^49^ reference material was generated by cold ablation of bulk polypropylene (PP30-SH-000155, GoodFellow).^50^ Polystyrene nanobeads were purchased from Polysciences Inc. (0.10 μm (00876), and 0.050 μm (15913) Polybead Microspheres, Polysciences Inc.)

### Sample Preparation

Human small intestinal organoids (HSIOs), purchased from the Human Organoid Innovation Hub (HOIH) at the University of Calgary, were cultured in Human IntestiCult Organoid Growth Medium (human) (06010, StemCell Technologies) according to the manufacturer’s protocol. Human small intestinal epithelial cells (HIEC-6, CRL-3266, ATCC) were expanded using Opti-MEM I Reduced Serum Medium (31985, Gibco) supplemented with 20 mM HEPES (07299, StemCell Technologies), 10 mM GlutaMAX (35050, Gibco), 4% fetal bovine serum (FBS) (A5209502, ThermoFisher Sci.) and 10 ng/mL of Epidermal Growth Factor (EGF, 236-EG-200, R&D Systems. 3D human skin models (HSMs) were prepared as described in previous work.^51^ Briefly, an ice-cold, acellular, collagen-based pregel solution was cast onto 12-well transwell inserts and then allowed to polymerize at 37 °C. A suspension of fibroblasts and monocytes in the same matrix solution was then layered on top and permitted to gel. After initial stabilization, culture media were added to both chambers and maintained to allow matrix contraction. Subsequently, keratinocytes were seeded onto the collagen layer and allowed to attach. After further incubation, the culture was transitioned to air–liquid interface conditions to promote epidermal differentiation, with regular media changes over 2 weeks.

For exposure to nanoscale plastics, NPPP-1 was diluted in HSIO or HIEC-6 cell-specific media to concentrations of 1 × 10^10^–1 × 10^12^ particles/mL, most commonly 1 × 10^11^ particles/mL (9.6 ± 0.9 μg/mL). Both HSIOs and HIEC-6 cells were passaged onto 12-well plates and then exposed to NPPP-1 for 92 h. HSM exposure was conducted using 1 mL of PS nanobeads in epidermalization media at a concentration of 1 × 10^11^ particles/mL (∼55 μg/mL) for 92 h. All the cultures and treatments were maintained in a cell culture incubator setup at 37 °C and 5% CO_2_. These concentrations were selected to ensure that the polymer infrared signal could be detected against the complex biological spectral background and to evaluate the sensitivity limits of the hyperspectral imaging and anomaly detection framework; they were not intended to represent environmentally realistic exposure levels.

After the exposure was completed, both HSIOs and HSMs were fixed in 4% paraformaldehyde (PFA) and embedded in optimal cutting temperature (OCT) compound (Fisher Scientific). A Leica CM1950 cryostat was then used to obtain 10 μm frozen sections. These sections were placed on IR-reflective slides (MirrIR, Kevley Technologies) and incubated at 37 °C for 5 min to promote adhesion. The slides were then washed in PBS at room temperature for 10 min to remove OCT. Finally, the slides were washed with Milli-Q water for 10 min and air-dried overnight. For HIEC-6, the cells were trypsinized (07901, StemCell Technologies) to obtain a single cell suspension, which was then washed with PBS, fixed with 4% PFA, and approximately 1 × 10^5^ cells were seeded onto IR-reflective slides using a Cytospin 4 (Fisher Scientific) at 1000 rpm for 5 min.

### Preparation of Polystyrene-Doped Matrigel Biomatrix Phantom Samples

Polystyrene (PS) nanoparticle suspensions were prepared using commercially available 50 nm carboxylated PS nanobeads. To generate biomatrix phantoms, Matrigel (356231, Corning) was diluted to 10% (v/v) in culture medium (DMEM/F-12, 21041, Gibco). PS particles were added to the diluted Matrigel at concentrations of 1 × 10^11^–1 × 10^13^ particles/mL (6.9–690 μg/mL). All mixing and dilution steps were performed on ice with prechilled pipet tips to prevent premature gelation. Samples were mixed gently by pipetting to minimize bubble formation. Prepared suspensions were subsequently deposited onto prewarmed low-e reflective glass microscope slides, allowed to gel at 37 °C for 30 min, and then dried for IR measurements. Care was taken to maintain consistent volumes and mixing conditions across samples to ensure comparable matrix composition and particle distribution. Carboxylated PS particles were used to promote homogeneous particle distribution within the matrix.

### QCL-IR Imaging

Infrared hyperspectral imaging was performed using a Bruker Hyperion II quantum cascade laser (QCL) infrared imaging microscope equipped with a 15×/0.4 NA objective in transflection geometry. The QCL source consisted of 4 tunable external-cavity lasers spanning 980–1900 cm^–1^, coupled to a room-temperature microbolometer detector with a 200-pixel × 200-pixel focal plane array. Hyperspectral images were recorded with a sampling interval of 2 cm^–1^ and collected by coadding 3 scans. Each acquisition produces a tile containing 40,000 spectra, each represented as a 460-length vector, and larger images were produced from multiple tiles.

### AFM Imaging

To confirm that NPs were present in the same tissue areas analyzed by QCL-IR, the slides were imaged with AFM. AFM topography images were recorded using NanoWizard II BioAFM (JPK Instruments, Berlin, Germany) mounted on an Olympus IX81 inverted microscope. AFM was performed in contact mode, and images were captured using DNP-S (Veeco/Bruker, CA) cantilever/tips with nominal spring constants of 0.06 N/m and a set point of 1 V. Following preliminary scans of larger areas (50 μm by 50 μm - 100 μm by 100 μm) for sample overview and guided by IR heatmaps, multiple smaller areas (5–20 μm) were recorded with a 512 pixel × 512-pixel resolution and a scan rate of ∼1 Hz. AFM images were processed and analyzed using JPK DP Processing (version 5.1.8).

### Data Preprocessing & Assembly

Spectra were preprocessed by baseline correction using the pybaselines package,^52^ typically with rubberband, or in some cases with iterative reweighted spline quantile regression (IRSQR).^53^ The primary training data set was established by the collection of 10 images of HSIOs, which were not exposed to NPs, consisting of 1.36 × 10^6^ spectra in total. Images were inspected for anomalous content that could contaminate the training data and excluded from the training set. Additional training sets specifically for HSMs and HIEC cells were tested, as was a combined data set from all sources. A separate training set of Matrigel biomatrix phantoms was used for models applied toward quantitative LOD estimations.

### Model Architecture, Training and Evaluation

A series of autoencoder models with varying complexity was used to reconstruct the original spectra and detect anomalies. These include a fully connected autoencoder (FC), a 1-D CNN autoencoder (CNN), and a hybrid CNN-encoder/FC-decoder (CNN-FC). Autoencoders function by reducing dimensionality to a set of compressed variables (a bottleneck) to generate representative features, and then re-expanding these to produce a reconstruction of the original data. The FC autoencoder performs this compression and decompression through 2 or more dense layers, in which each node in a layer receives input from all nodes in the previous layer. In contrast, a CNN autoencoder uses sliding convolutional filters (kernels) to produce parallel feature maps (channels), which are downsampled by the kernel stride. This produces richer feature maps that account for feature locality and shifting. A hybrid architecture combining the rich feature extraction of the CNN encoder with an FC decoder to provide simpler global reconstruction mapping was employed as a balanced compromise.

Final model architectures are shown in Figure 1, with dimensions determined through hyperparameter tuning (Table S1). All models used 1D spectra as inputs, with the final output matching the initial vector length of the recorded IR spectra. Leaky RELU activation functions were used between layers, except for the final layers of the encoder and decoder, respectively.

**Figure 1:**
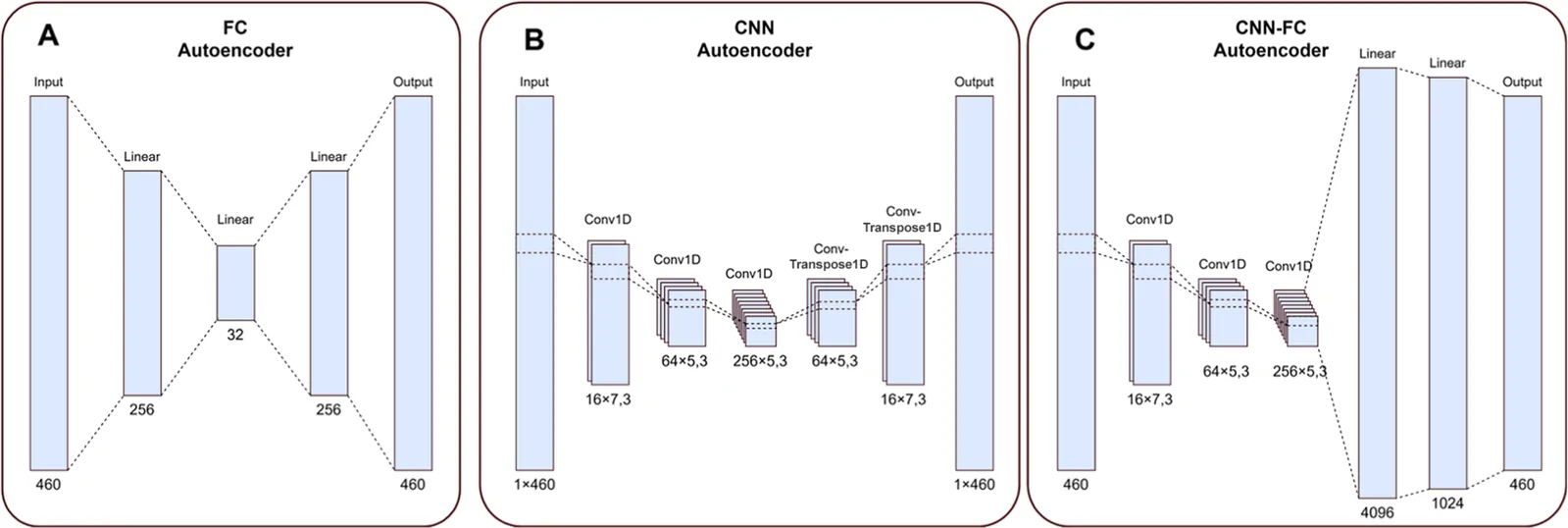
Neural network architectures of the autoencoder models used in this study. (A) Fully connected (FC) network, with input dimensions and layer sizes indicated below each layer. (B) Convolutional neural network (CNN), with dimensions labeled as channels × kernel, stride. (C) Autoencoder combining a CNN encoder with an FC decoder.

Model training was performed using a custom loss function that evaluated the mean squared error between the input and output spectra and their first derivatives, with the derivative contribution weighted by the hyperparameter γ (eq 1). This was performed as the derivative spectra can be a more accurate assessment of spectral similarity, being less influenced by noise and baseline drift. Since absolute absorbance values of the spectra are not preserved in the derivative, the loss function incorporates the combination of the original and derivative error.

1 $$Loss=\frac{1}{n}\sum_{k=1}^{n}{\left(x_{k}-{\mathrm{x̂}}_{k}\right)}^{2}+\gamma {\left(\frac{dx_{k}}{dy}-\frac{d{\mathrm{x̂}}_{k}}{dy}\right)}^{2}$$

Model performance was evaluated by computing the overall reconstruction error as the mean of the sum of absolute differences between all input and output points in an image (eq 2). Hyperparameter tuning was performed using a test image comprising a control sample separate from the training data, and a validation image consisting of a sample exposed to 1 × 10^11^ particles/mL NPPP-1, which contained detectable levels of plastics over a large area of the image. The goal during tuning was to minimize the reconstruction error on the test and validation samples, while maximizing the reconstruction error of the characteristic PP C–H bends at 1378 cm^–1^ and 1456 cm^–1^ on the validation sample. Models were scored on their relative ability to meet both criteria on the validation image (Table S1). Hyperparameters were evaluated using a grid search method. Evaluated hyperparameters included layer size and depth for fully connected layers; channels, strides, and kernels for the CNN layers. For all models, hyperparameters included the learning rate, the addition of dropout layers to the encoder, and the value of γ. All models were trained with 5 epochs. Full details are provided in the Supporting Information (Table S1).

2 $$reconstruction\;error=\frac{1}{n}\sum_{k=0}^{n}\sum_{i=0}^{m}{\left|x_{i}-{\mathrm{x̂}}_{i}\right|}_{k}$$

### Guided Residual Anomaly Detection

The residual (error) spectrum was calculated in two ways. One method was to take the difference between the input and reconstructed spectra. A second method, less prone to high-frequency noise, was calculated as the difference between the first derivatives of the input and reconstructed spectra, then restored using cumulative trapezoid integration. Anomaly detection is performed through either a guided or unguided approach. In the unguided case, the Euclidean distance between the input and reconstructed spectra is computed, and its inverse is used to generate a heatmap. In the guided case, a peak corresponding to a characteristic signal of the target polymer was targeted for integration, and integration was performed by subtracting a linear baseline over the specified integration region and applying the trapezoidal rule. The resulting integration can be displayed as a heatmap. An example that displays all processing steps, from raw data to the polymer-specific heatmap, is provided in Figure S1 of the Supporting Information.

### Software and Platform

Python 3.13 was used to preprocess the spectra. All models were built using PyTorch (v2.9). Models were trained on a desktop computer with an Intel i7–14700k CPU, an NVIDIA GeForce RTX 4060 GPU, and 32 GB of DDR5 RAM, running Windows 11.

## Results & Discussion

### Guided Anomaly Detection - Approach and Validation

Training and testing data sets were generated from control samples that were not exposed to NPs. These consisted of organoids sectioned and fixed onto IR-reflective microscope slides, as well as cells deposited via cytospinning. The spectral data of these images can be segmented into baseline regions devoid of material and regions corresponding to organoid or cellular structures (Figure 2A,B). Figure 2A displays randomly sampled spectra from a representative control organoid. Figure 2B overlays total infrared absorbance onto a visible image of the organoid, providing spatial context. The spectra exhibited characteristic signatures of biological samples,^25^ with the most prominent features being the amide I and II bands at approximately 1660 cm^–1^ and 1550 cm^–1^, respectively, followed by C–H vibrations at approximately 1450 cm^–1^ and 1360 cm^–1^, primarily associated with lipids and other molecules. Additional absorptions arising from carbonyls, phosphates, and other functional groups occur outside this region. However, the aforementioned region is of interest because it overlaps with common vibrational modes of polymers.

**Figure 2:**
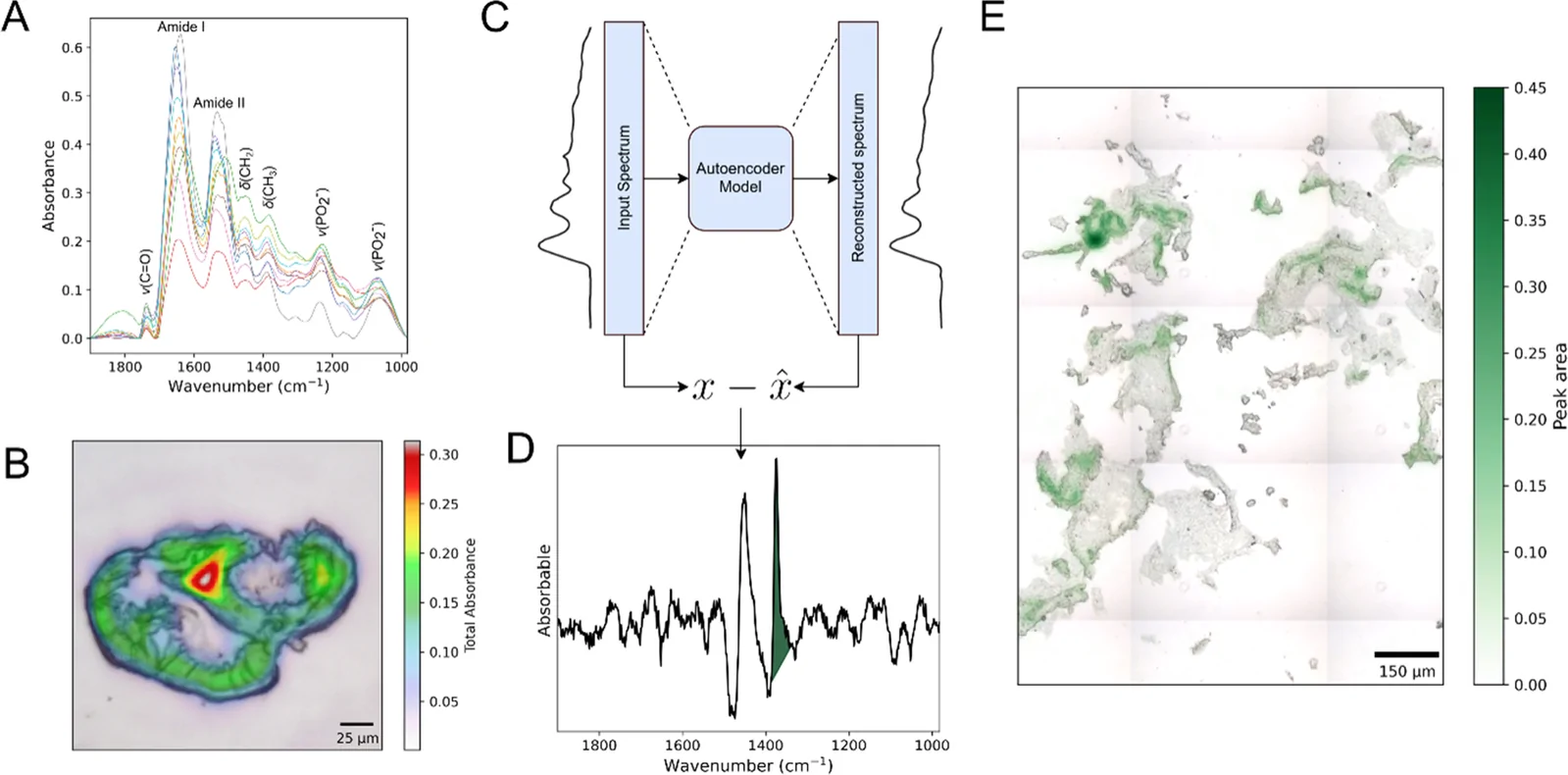
Autoencoder-based residual analysis enables detection and visualization of nanoscale polypropylene (PP) in hyperspectral IR data. (A) Randomly sampled IR spectra showing typical biological signatures, extracted from the hyperspectral IR image displayed in (B), which presents a total absorbance map of a representative control sample. (C) Schematic of the autoencoder-based residual anomaly detection workflow. An input spectrum is fed into the autoencoder, which outputs a reconstructed spectrum. (D) Residual spectrum obtained by subtracting the reconstructed spectrum from the input spectrum, highlighting discrepancies; reconstruction errors are greatest in regions containing nanoscale plastics. (E) Heatmap generated by integrating a region of interest in the anomalous residual signal, visualizing nanoscale PP accumulation.

To clearly distinguish overlapping signatures from biomolecules and polymers, the biological FTIR contribution must be effectively removed. Due to the inherent variability in biological samples, spectral peaks are broad and vary in both position and intensity, as evident in Figure 2A. Thus, simple subtraction of a single reference spectrum is insufficient. Instead, a method capable of dynamically fitting the biological matrix to each individual sample spectrum is required for accurate background removal.

This was accomplished by using autoencoder models trained exclusively on control samples not exposed to NPs to reconstruct the biological spectrum. In samples containing NPs, the residual between the original and reconstructed spectra enables isolation of any significant spectral components that are not endogenous to the cellular matrix (Figure 2C,D). If the target polymer is known, the residual result can be directly visualized by integrating a region of interest (ROI) corresponding to a characteristic vibrational band, such as the 1378 cm^–1^ C–H bending vibration for polypropylene, producing a heatmap of the target content (Figure 2E).

This guided strategy differs from conventional residual anomaly detection, in which a discriminative metric, such as the Euclidean or Mahalanobis distance, is used to quantify the overall difference between the original and reconstructed spectra or images.^54^ Instead, the guided strategy focuses on anomalies in a specific, predetermined range. Unguided metrics capture total reconstruction error and, therefore, are prone to highlighting nonspecific anomalies, including artifacts from imperfect reconstructions or unrelated chemical species. While in some applications this broader sensitivity may be beneficial, the ability to discriminate for specific chemical species can be advantageous. For example, Figure 3 shows contrasting heatmaps generated via anomaly detection using Euclidean distance (Figure 3A) and guided anomaly detection generated by integrating a target ROI associated with PP IR absorption (Figure 3B). When reconstruction error is calculated using Euclidean distance, regions where the residual spectrum is dominated by reconstruction noise, local variations in the biological matrix, or chemical species not associated with polymers may appear as high-priority anomalies on the map (Figure 3C). However, limiting the analysis to a chemically specific ROI (i.e., the 1378 cm^–1^ vibrational band) enables the guided approach to produce a spatial distribution with residuals that more closely match the PP spectral signature.

**Figure 3:**
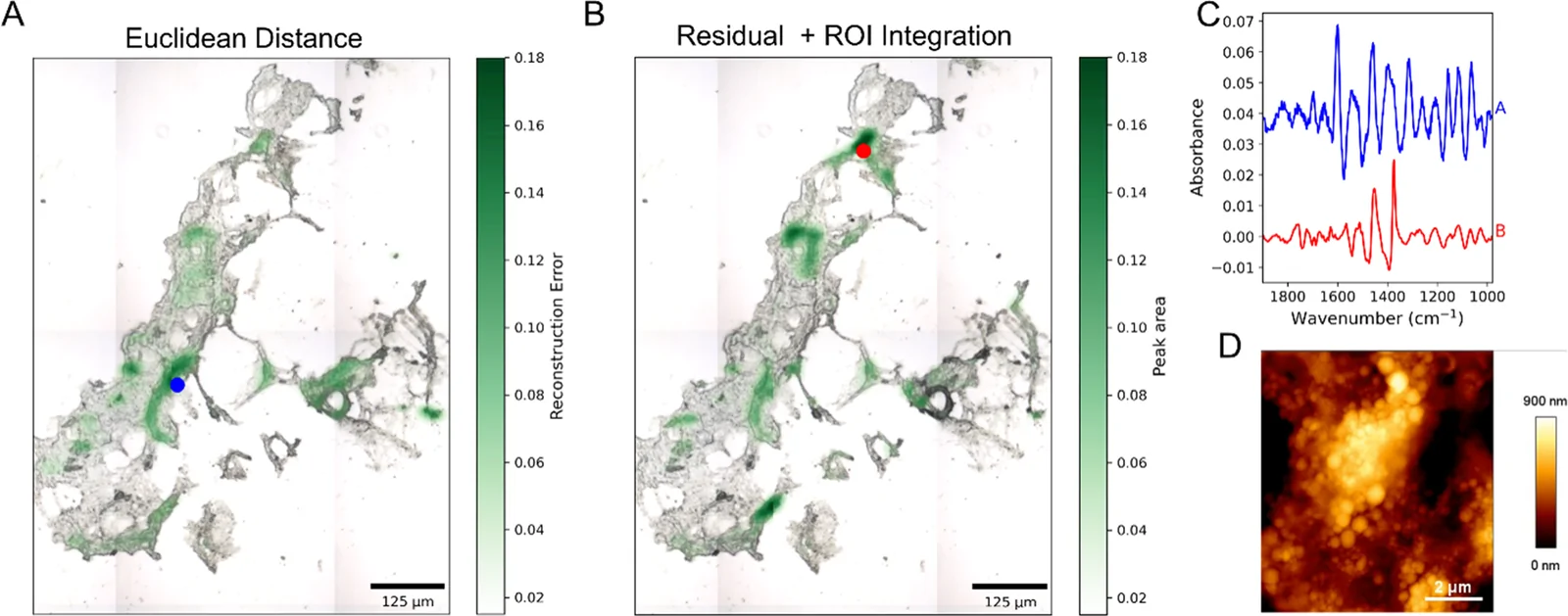
CNN-FC–based anomaly detection highlights NPPP-1 accumulation in human small intestinal organoids (HSIO) samples. (A) Heatmap generated from CNN-FC model using the Euclidean distance, displaying maximal reconstruction error. (B) Heatmap of the same sample generated by integrating the 1378 cm^–1^ mode of polypropylene in the residual error spectrum. The image in B shows greater specificity for NPPP-1 accumulations. (C) Residual spectra extracted from the strongest points in each heatmap, with ROI-guided detection (red) showing two peaks resembling the spectral signature of polypropylene. In contrast, the unguided approach by Euclidean distance (blue, offset) shows a contribution from reconstruction noise. (D) AFM height image of a hotspot of NP accumulation, confirming the presence of NPPP-1 aggregates at locations identified by the guided approach.

The presence of the target plastic can be confirmed by examining residual spectra extracted from high-intensity regions in the guided map. For example, in samples exposed to NPPP-1, a spectrum containing peaks at 1456 cm^–1^ and 1378 cm^–1^ can be recovered, agreeing with the strongest resonances in the reference spectrum. Follow-up imaging with atomic force microscopy (AFM) provides secondary confirmation, revealing that NPPP-1 particles accumulate in regions associated with the guided-detection map (Figure 3D). AFM imaging confirms the presence and spatial distribution of nanoscale particles in the exposed biological samples. Polymer identification is obtained from the infrared spectral signatures extracted from hyperspectral data using the anomaly detection framework.

### Model Design and Training Considerations

A number of different model architectures were developed and evaluated for biological sample anomaly extraction (Figure 1): (i) a fully connected (FC) autoencoder, (ii) a convolutional neural network (CNN) based autoencoder, and (iii) a hybrid, convolutional encoder, fully connected decoder (CNN-FC) architecture. All models were trained to minimize the reconstruction error on hyperspectral images of control samples. During hyperparameter tuning, two competing objectives were considered: (1) to minimize overall reconstruction error, and (2) to maximize reconstruction errors at 1456 cm^–1^ and 1378 cm^–1^, corresponding to the primary C–H vibrational bands of polypropylene, in a validation data set weakly containing these signals. The final architectures are outlined in the methods section and Table S1.

The FC autoencoder served as the initial basis for implementation. During hyperparameter tuning, this model performed best with a relatively shallow architecture (the encoder only 2 layers deep) and a relatively wide bottleneck of 32 nodes. This indicates that the data could be reconstructed accurately by a relatively shallow network, without requiring the additional hierarchical feature mapping provided by a deeper network. A wide bottleneck retained more information and achieved lower reconstruction error than the severe compression imposed by a narrower bottleneck. However, a principal limitation was residual noise arising from poor reconstruction in certain regions of the spectrum, particularly near the strong amide II peak. A more structured CNN model was explored to improve reconstruction quality and signal-to-noise. Compared to FC architectures, convolutional models encode locality and hierarchical structures. Thus, more capable of recognizing structures such as peaks and shoulders while being more robust to their shifting. This improves their potential for reconstruction quality. As reconstruction quality improved with the CNN autoencoder, it exhibited a different limitation: reducing the signal from target polymers in the residual spectrum, as shown in Figure 4B. This runs contrary to the classical definition of overfitting (i.e., poor generalization). Here, the model generalizes too effectively to new data, reconstructing polymer signatures along with the biological matrix, thereby diminishing anomaly contrast. While reconstruction error was minimized, anomaly detection was compromised.

**Figure 4:**
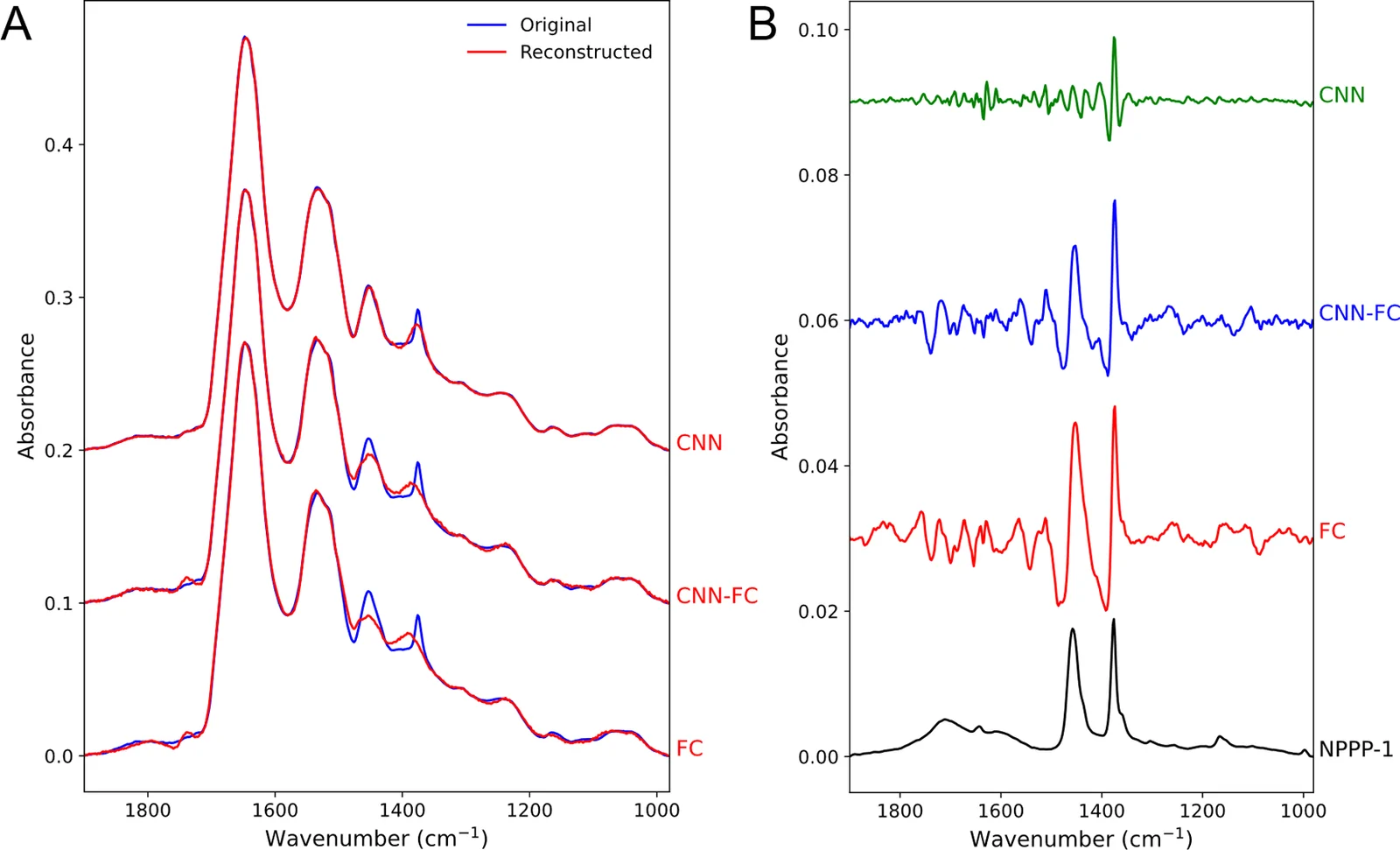
A comparison of spectral reconstruction and residual analysis highlights how different models capture the NPPP-1 chemical signatures. (A) Original and reconstructed spectra in an NPPP-1-containing area as evaluated by three different models. (B) Residual spectra, with a reference NPPP-1 spectrum provided, showing that the FC and CNN-FC autoencoders reproduce strong peaks at 1456 cm^–1^ and 1378 cm^–1^ (PP) but with higher residual noise due to reconstruction errors, whereas the CNN model reduces noise but overfits the polypropylene peaks, affecting the identifiable chemical signature.

A compromise solution combined the representational capacity of a convolutional encoder with a less expressive fully connected decoder. This CNN-FC architecture preserved deep spectral encoding while limiting decoder overfitting. This hybrid model improved reconstruction quality relative to the FC architecture (Table S2) and retained anomalous polymer features better than the fully convolutional architecture.

The models presented here are basic neural network architectures applied toward anomaly detection in hyperspectral imaging applications. As models become more complex, their hyperparameter spaces grow, making optimization for a specific application more challenging. In this case, hyperparameter tuning was performed to minimize the overall reconstruction error while maximizing the reconstruction error for polypropylene. By these criteria, the CNN architecture performed best, minimizing overall reconstruction error while maximizing the error at 1378 cm^–1^ (Figure 4B). However, the reconstructed spectra failed to retain interpretable polymer spectral features of the anomalous species. The FC and CNN-FC architectures appropriately preserved spectral information but at the cost of increased residual noise. Further optimization could incorporate chemically informed loss functions or anomaly preserving constraints during training. Other model architectures, such as variational autoencoders,^47^ generative adversarial networks,^45^ or long short-term memory networks,^55^ may further improve reconstruction quality and generalization, reduce the effect of anomaly contamination in training data, and improve the overall efficiency of this residual anomaly detection approach.

Data preprocessing also has a noticeable effect on model training and performance. Data preprocessing consisted of applying baseline correction to all spectra in the data set. Normalization, a step typically deemed important in preparing data sets for machine learning tasks, was not performed as the nature of IR absorbance measurements and the sample preparation constrain absorbance values to roughly between 0 and 1. The application of baseline correction simply pins the floor of absorbance values to 0; thus, a relatively simple rubberband algorithm was used to anchor absorbance minima at 0, allowing models to learn higher-order baseline variability.^56^ More aggressive preprocessing, such as through iteratively reweighted spline quantile regression (IRSQR),^57^ reduced degrees of freedom in the data, accelerated convergence, and lowered reconstruction noise. Similar to the CNN model, excessive preprocessing increased the risk of anomaly suppression (Figure S2). Consequently, the extent of preprocessing must be balanced against the model’s representational capacity to preserve relevant residual features.

### Application to Nanoscale Plastics Detection

The technique presented here was primarily applied to detect nanoscale plastics in biological samples comprising 2D monolayer cell cultures or 3D organoids. These cultures were exposed to nanoscale polypropylene (NPPP-1) or polystyrene at various concentrations (1 × 10^10^–1 × 10^12^ particles/mL), and uptake of particles was visualized by QCL-IR combined with a guided anomaly detection-based approach. Heatmaps signifying NP accumulation were observed in multiple systems. In the HIEC-6 2D cultures (Figure 5A), intracellular accumulation was observed in a subset of cells. This heterogeneity in intracellular particle burden at the single cell level indicates differential uptake behavior, potentially reflecting variations in endocytic activity or membrane dynamics, both of which have been associated with downstream oxidative stress and inflammatory responses following MNP exposure.^58^ HSIOs (Figure 5B) showed accumulations of NPPP-1 localized both external to and internally within organoid structures. Spatial discrimination between particle accumulation external to the epithelial barrier and within the organoid lumen or epithelial layer provides a mechanistic context for barrier interaction and potential translocation, which are key determinants of toxicological impact in gastrointestinal exposure models.^59^ HSMs (Figure 5C) were exposed to polystyrene nanobeads, a common commercially available material used in the study of nanoplastics toxicity, which accumulated primarily in the epidermis with limited dermal penetration. Given that the HSM recapitulates key architectural features of stratified human skin, including a differentiated epidermal barrier and an underlying dermal compartment, layer-specific localization provides important context for assessing dermal exposure.^51^ Epidermal confinement versus dermal penetration may influence local inflammatory signaling, immune cell activation, and the potential for systemic distribution.^60^ The ability to resolve particle distribution across defined tissue compartments, therefore, strengthens the interpretation of biological interaction beyond bulk exposure metrics. In all these cases, QCL-IR imaging confirmed NP uptake. It localized their presence, demonstrating applicability across a variety of biological systems and analytes (polypropylene and polystyrene), giving confidence that the method could be further employed in other settings. Moreover, beyond confirming the presence of particles, spatial mapping of nanoscale plastics provides a framework for linking physical particle burden and compartment-specific accumulation to established toxicological pathways, supporting future integration with molecular and functional readouts for comprehensive ecological and biological impact assessment.

**Figure 5:**
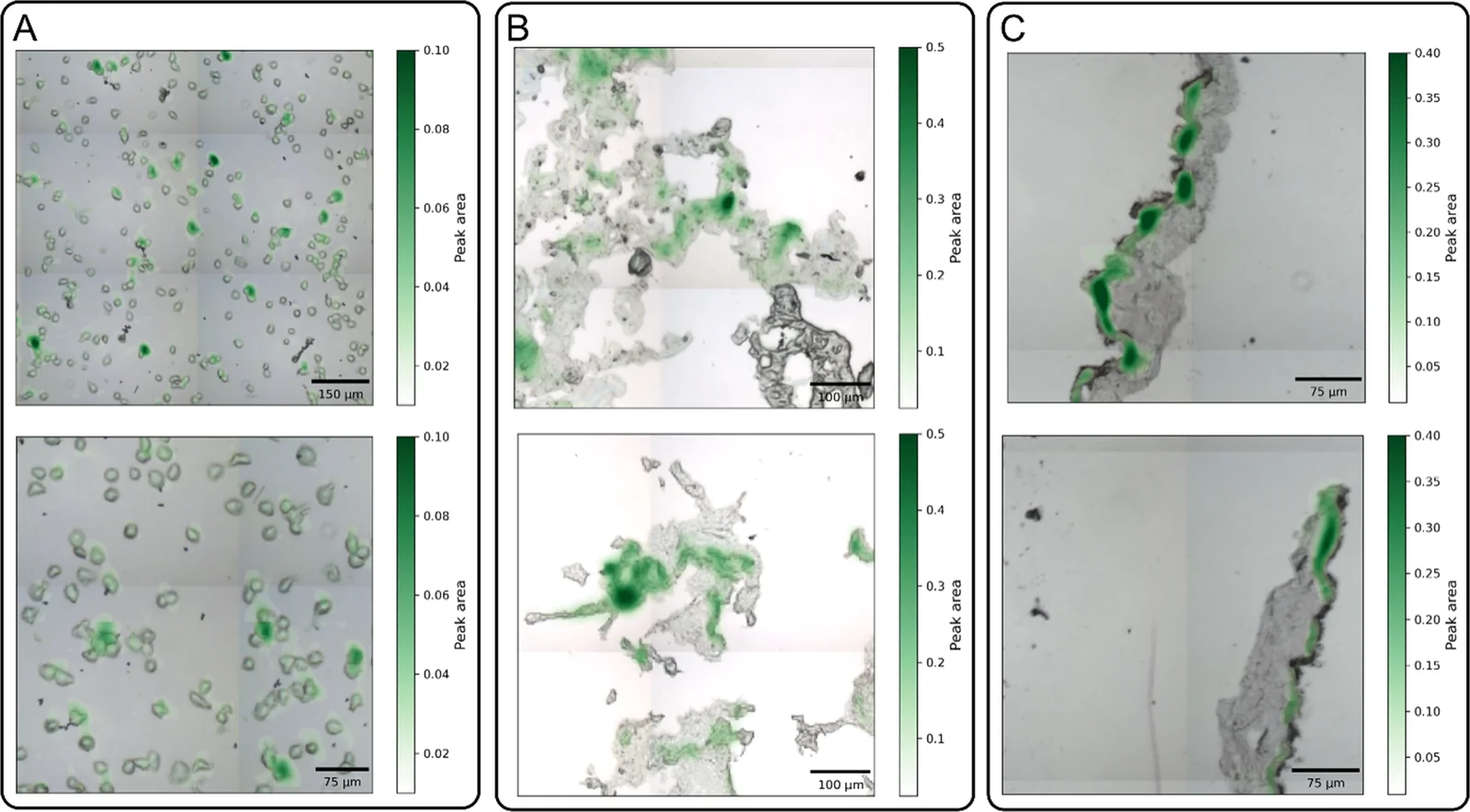
Guided anomaly detection using a CNN-FC model trained on a generalized data set reveals system-specific nanoscale plastic uptake and localization across different cellular models. (A) Human intestinal epithelial cell (HIEC) 2D monolayer cultures exposed to 1 × 10^11^ particles/mL NPPP-1. (B) Human small intestinal organoids (HSIOs) exposed to 1 × 10^11^ particles/mL NPPP-1. (C) Human skin model (HSM) exposed to 1× 10^11^ particles/mL PS nanobeads.

The employment of this method across a variety of systems can take two approaches, depending on the available data. Two training strategies were evaluated: (i) System-specific training using control images from the same 2D or 3D samples; (ii) A generalized broad training using aggregated control data sets. Using a system-specific, specialized training set reduces reconstruction errors in control and validation data sets; the signal-to-noise ratio in NP-containing regions shows minimal improvement compared to a generalized training set (Figure S3). Either approach proved to be a valid strategy. Still, system-specific training sets can offer superior reconstruction quality when sufficient data are available, whereas a generalized data set can be readily applied when several systems are being studied. A more consequential factor was the overall size of the training data used. Typically, in machine learning workflows, larger training data sets are used to reduce overfitting, improving models’ ability to generalize to new data and their overall performance. However, increasing the data set size can also lead to overgeneralization, overfitting to anomalous data, and reduced anomaly detection (Figure S4), thereby decreasing overall model performance (Table S3). A balance between model capacity and training data size is necessary to maximize the number of identifiable residual features.

### Limit of Detection

Detection sensitivity is governed by both the efficiency of residual background removal and the physical limits of QCL-IR microscopy. Hyperspectral images acquired during this work were obtained in the wavenumber range of 980–1900 cm^–1^. IR microscopy is often stated to be diffraction-limited for particle sizes >10–20 μm,^29^ though that applies to the visualization of isolated single particles. For submicron and nanoscale plastics, detection is governed by the absorbance arising from local particle concentrations at any given point in the hyperspectral image; only accumulations above a minimum threshold density will be detectable. Because nanoplastics are far below the diffraction-limited spatial resolution of MIR imaging, detection depends on accumulating sufficient particle mass within each hyperspectral pixel to produce measurable absorbance. Under environmentally realistic exposure conditions, where particles are more sparsely distributed, the resulting polymer absorbance may fall below the instrumental detection threshold. Empirically, approximately 1 × 10^11^ particles/mL represented an optimal exposure concentration. Lower exposures frequently yielded undetectable accumulation, whereas 1 × 10^12^ particles/mL produced strong absorbance, reducing the need for guided residual processing. Examples of these exposures are provided in the Supporting Information (Figure S5).

These exposure levels do not provide information on the local concentrations observed in the hyperspectral images. To provide a quantitative estimate of detectable nanoscale plastic concentrations, a biological matrix phantom was created using extracellular matrix (ECM) loaded with known concentrations of 50 nm carboxylated polystyrene beads. The carboxylated surface of nanobeads ensured uniform dispersal in the ECM. A CNN-FC model was trained on control ECMs containing no nanobeads; all spectra were normalized to the Amide I stretch of the ECM to account for heterogeneous deposition of matrix material. Figure 6A shows the resulting peak height at 1494 cm^–1^ for the residual spectra obtained from these measurements. The noise level (σ) was established as the average RMS noise in the residual spectra of a control ECM image not included in the training data. This indicates the nominal detection limit (3σ) of 50 nm polystyrene nanobeads when the local concentration is ∼1% (mass/mass) in dried biological material, where total biological material was estimated as the sum of the protein content in Matrigel, as well as the amino acid and glucose content of the dilution media. It was observed that reconstruction noise scaled with the polymer signal in the residual spectra (Figure 6C), thereby limiting the signal-to-noise ratio as polymer content increased, yet still allowing faint polymer signatures to be observed near the noise level recorded in low polymer-content samples. The model’s fitting quality establishes the noise floor, influencing the limit of detection. Near this detection limit, IR peaks from polymers are faint enough to be indistinguishable from the background in the original spectra (Figure 6B); the residual anomaly detection process reveals polymer-related signals that may otherwise be unobservable (Figure 6C). This example represents an idealized scenario with consistent, uniform data and a well-fitting model, yet observations were roughly maintained in samples with a narrow plastic content range and a more heterogeneous ECM (Figure S6). In biological systems, nanoscale plastics may appear as aggregates, agglomerates, or intracellular clusters formed during uptake and transport processes. Such localized accumulations increase the effective polymer mass, thereby enhancing the likelihood of detectable infrared absorption signals.

**Figure 6:**
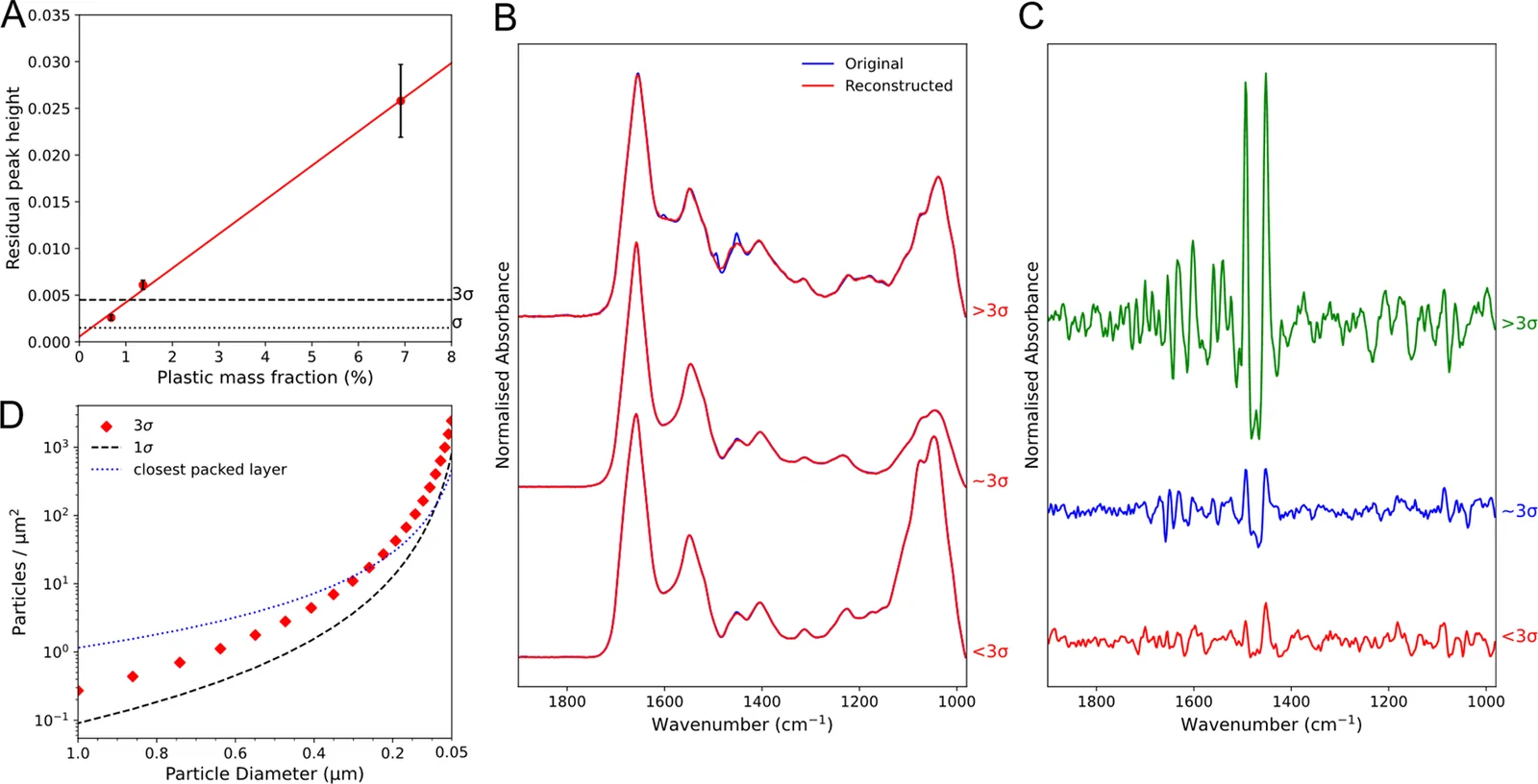
Detection limits and IR spectral analysis of 50 nm polystyrene (PS) in extracellular matrix. (A) Detectable limit of 50 nm polystyrene relative to the total mass of extracellular matrix. The formal limit of detection is established as 3σ, where σ is the RMS noise of the residual spectrum of a control ECM. The corresponding mass density of PS is ∼1% of the dried biological material. (B) Sample original and reconstructed IR spectra from corresponding mass fractions of PS. At low PS content, PS specific IR signatures are not distinguishable in the original spectra. (C) The residual spectra, where even below the 3σ threshold, the 1452 cm^–1^ and 1494 cm^–1^ bands are visible. (D) Theoretical limit of detection of local number density for submicron PS based on Mie scattering. Particle densities at the red points (3σ) represent the estimated limit of detection, with the noise floor (σ) denoted by the black line. The blue trace represents a closest-packed monolayer of particles, indicating the point at which submonolayer concentrations become undetectable.

A theoretical estimate of the local particle number density was established using absorption cross sections derived from Mie scattering calculations (Figure 6D), based on published complex refractive indices of polystyrene.^61^ The particle density required to reach the absorbance equal to 3σ was determined at the C–H bending vibration (1494 cm^–1^). Figure 6D shows the theoretical minimum detectable particle density as a function of particle size, from 50 to 1000 nm. Note that this minimum also assumes that the signal in question is not diminished in the model’s reconstruction of the spectra; the noise level was established from the RMS noise in control sample residual spectra (Table S2). For sufficiently small particle sizes (<200 nm), the theoretical detection limit approaches the coverage corresponding to a close-packed two-dimensional (2D) monolayer, indicating that detectable infrared absorbance arises only when the local polymer mass within a diffraction-limited sampling region exceeds that of a single densely packed layer. For particle sizes greater than 200 nm, the detection limit falls below the monolayer threshold, meaning that submonolayer surface densities are potentially detectable. This transition is illustrated in Figure 6D, where the blue dotted line intersects the red data points at approximately 250 nm, marking the size at which the detection limit converges toward a close-packed 2D layer; below this size, detection requires more than a single particle layer within the sampling area. These results were obtained using polystyrene spheres as a model system. Differences in particle composition and morphology are expected to alter absorption cross sections and may shift the absolute detection limits. This estimate indicates that detection arises from collective absorption by hundreds to approximately 1000 nanoparticles within a diffraction-limited sampling region; isolated single nanoparticles cannot be detected under these conditions.

Techniques such as AFM-IR and optical photothermal infrared (O-PTIR) spectroscopy provide substantially higher spatial resolution for vibrational analysis of nanoscale materials, although typically over smaller fields of view than hyperspectral infrared microscopy. The anomaly detection framework presented here may therefore complement such techniques and could be integrated with higher-resolution vibrational imaging methods in future studies.

Future improvements in detection sensitivity may also arise from advances in infrared source technology and noise-reduction strategies. For example, quantum-optical approaches such as squeezed-light generation have been shown to reduce measurement noise below the standard quantum limit in optical spectroscopy, potentially improving the effective signal-to-noise ratio in absorption measurements. In principle, such approaches could enhance the detectability of weak polymer absorption signals in hyperspectral infrared imaging, although their implementation in broadband mid-infrared microscopy remains an area of ongoing research.

Although environmental exposure levels may be low during individual exposure events, repeated exposure and biological retention within tissues or cellular compartments may lead to localized accumulation of nanoscale plastics. Such accumulation can produce sufficient polymer mass to generate detectable infrared signals within a hyperspectral imaging pixel. Such accumulation processes are particularly relevant from a risk-assessment perspective and may generate concentrations detectable by hyperspectral infrared imaging. When particles are extremely dilute and spatially dispersed, their combined absorbance falls below the instrumental detection threshold.

## Conclusion

Residual anomaly detection using autoencoder networks enables the extraction of the infrared spectral signature of nanoscale plastic accumulations, which can be used to visualize their localization within cells, tissues, and other complex biological matrices. By “learning” the spectral characteristics of the biological matrix signature, this technique enables effective background subtraction. A central finding is that optimal anomaly detection does not correspond to minimal reconstruction error. Architectures with high representational capacity, such as fully convolutional autoencoders, can suppress anomalous polymer signatures by reconstructing them along with the biological matrix. In this application, excessive reconstruction fidelity reduces chemical contrast. Fully connected and hybrid CNN–FC architectures provided a more appropriate balance between matrix reconstruction and anomaly preservation. The guided anomaly detection approach integrates chemically defined regions of interest within residual spectra and improves specificity compared to global distance-based metrics. Rather than highlighting regions of maximal reconstruction discrepancy, the guided approach selectively emphasizes signals consistent with known polymer vibrational modes, enabling chemically interpretable heatmaps of nanoparticle accumulation. Applying the guided anomaly detection technique to QCL-IR imaging enabled label-free visualization of nanoscale accumulations of polypropylene and polystyrene in a variety of 2D and 3D cell cultures. An empirical analysis using a biological matrix phantom, together with a theoretical analysis based on residual noise and Mie scattering, establishes quantitative constraints on particle detectability in terms of local mass fraction and number density, clarifying the physical limits of the approach.

More broadly, this work illustrates that anomaly detection in chemically complex hyperspectral data sets requires deliberate control of model capacity. Chemically informed residual interrogation is critical for preserving target spectral signatures. This approach is extensible to other hyperspectral imaging modalities with greater spatial resolution, including Raman, O-PTIR or near-infrared spectroscopy and imaging, where selective extraction of target spectral signatures from heterogeneous backgrounds is required. Because the anomaly detection framework operates on spectral data rather than a specific imaging modality, the same approach could in principle be applied to hyperspectral data sets acquired using higher-resolution vibrational techniques such as O-PTIR or AFM-IR. Integration with such methods may enable detection of smaller particle clusters and potentially improve nanoscale chemical localization. Beyond nanoscale plastic detection, this framework applies to any scenario in which a defined spectral signature must be resolved against a structured and variable background, such as trace contaminant detection, drug localization studies, pathological spectral mapping, and phase discrimination in heterogeneous materials. As hyperspectral imaging continues to expand across biological and materials sciences, such chemically informed residual frameworks will become increasingly important for translating complex spectral data sets into interpretable, spatially resolved chemical information.

## Supplementary Material



## Data Availability

Python code for training and evaluating the machine learning models used in this work is available as Jupyter notebooks at 10.4224/40004000. Associated data is available upon request.

## Notes

Details of hyperparameter fitting. Spectra and produced heatmaps for: demonstrating the full process from data preprocessing to model fitting and heatmap production; the effect of baseline correction on model fitting; the effect of generalized and system specific model training data on model fitting; the effect of training data size on model fitting; comparison of anomaly detection in high and ultralow plastic content samples; alternative LOD calibration at a narrow plastic content range (PDF)

## References

[1] MacLeod M., Arp H. P. H., Tekman M. B., Jahnke A. (2021). The Global Threat from
Plastic Pollution. Science.

[2] Rhodes C. J. (2018). Plastic
Pollution and Potential Solutions. Sci. Prog..

[3] Shahul
Hamid F., Bhatti M. S., Anuar N., Anuar N., Mohan P., Periathamby A. (2018). Worldwide Distribution and Abundance
of Microplastic: How Dire Is the Situation?. Waste Manag. Res..

[4] Shen M., Zhang Y., Zhu Y., Song B., Zeng G., Hu D., Wen X., Ren X. (2019). Recent Advances
in Toxicological
Research of Nanoplastics in the Environment: A Review. Environ. Pollut..

[5] Sangkham S., Faikhaw O., Munkong N., Sakunkoo P., Arunlertaree C., Chavali M., Mousazadeh M., Tiwari A. (2022). A Review on Microplastics
and Nanoplastics in the Environment: Their Occurrence, Exposure Routes,
Toxic Studies, and Potential Effects on Human Health. Mar. Pollut. Bull..

[6] Schröter L., Ventura N. (2022). Nanoplastic Toxicity:
Insights and Challenges from
Experimental Model Systems. Small.

[7] Li X., Chen Y., Zhang S., Dong Y., Pang Q., Lynch I., Xie C., Guo Z., Zhang P. (2023). From Marine
to Freshwater Environment: A Review of the Ecotoxicological Effects
of Microplastics. Ecotoxicol. Environ. Saf..

[8] Alimba C. G., Faggio C., Sivanesan S., Ogunkanmi A. L., Krishnamurthi K. (2021). Micro­(Nano)-Plastics in the Environment
and Risk of
Carcinogenesis: Insight into Possible Mechanisms. J. Hazard. Mater..

[9] Covello C., Vincenzo F. D., Cammarota G., Pizzoferrato M. (2024). Micro­(Nano)­Plastics
and Their Potential Impact on Human Gut Health: A Narrative Review. Curr. Issues Mol. Biol..

[10] Paul M. B., Böhmert L., Hsiao I.-L., Braeuning A., Sieg H. (2023). Complex Intestinal and Hepatic in Vitro Barrier Models Reveal Information
on Uptake and Impact of Micro-, Submicro- and Nanoplastics. Environ. Int..

[11] Feng Y., Tu C., Li R., Wu D., Yang J., Xia Y., Peijnenburg W. J. G. M., Luo Y. (2023). A Systematic Review of the Impacts
of Exposure to Micro- and Nano-Plastics on Human Tissue Accumulation
and Health. Eco-Environ. Health.

[12] Zhang H.-J., Li S., Wang X.-L., Zhang K.-D., Fang H.-T., Wu X., Huang Z., Jiang W., Yang L., Tan Q.-G., Pan B., Ji R., Wang P., Xing B., Miao A.-J. (2025). Size-Dependent
Translocation of Polystyrene Nanoplastics across Biological Barriers
in Mammals. Nat. Commun..

[13] Hua X., Wang D. (2022). Cellular Uptake, Transport, and Organelle Response
after Exposure
to Microplastics and Nanoplastics: Current Knowledge and Perspectives
for Environmental and Health Risks. Rev. Environ.
Contam. Toxicol..

[14] Du B., Li T., He H., Xu X., Zhang C., Lu X., Wang Y., Cao J., Lu Y., Liu Y., Hu S., Li J., Li L., Shi M. (2024). Analysis of Biodistribution
and in Vivo Toxicity of Varying Sized Polystyrene Micro and Nanoplastics
in Mice. Int. J. Nanomedicine.

[15] Vleeming J., Laan S. N. J., Bosker T. (2026). Uptake and Translocation
of Polystyrene
Nanoplastics in Edible Plants via Root and Foliar Exposure: A Qualitative
Imaging-Based Study. Environ. Pollut..

[16] Akhatova F., Ishmukhametov I., Fakhrullina G., Fakhrullin R. (2022). Nanomechanical
Atomic Force Microscopy to Probe Cellular Microplastics Uptake and
Distribution. Int. J. Mol. Sci..

[17] Yang L., Tang B. Z., Wang W.-X. (2023). Near-Infrared-II In Vivo Visualization
and Quantitative Tracking of Micro/Nanoplastics in Fish. ACS Nano.

[18] Zhang H.-J., Zhou H.-R., Pan W., Wang C., Liu Y.-Y., Yang L., Tsz-Ki Tsui M., Miao A.-J. (2023). Accumulation of
Nanoplastics in Human Cells as Visualized and Quantified by Hyperspectral
Imaging with Enhanced Dark-Field Microscopy. Environ. Int..

[19] Aramendia J., García-Velasco N., Amigo J. M., Izagirre U., Seifert A., Soto M., Castro K. (2024). Evidence of Internalized
Microplastics in Mussel Tissues Detected by Volumetric Raman Imaging. Sci. Total Environ..

[20] Gruber E. S., Karl V., Duswald K., Bhamidipalli M. S., Schlederer M., Limberger T., Kopatz V., Teleky B., Kenner L., Brandstetter M. (2025). Method for
Label-Free & Non-Destructive
Detection of Microplastics in Human Formalin-Fixed Paraffin-Embedded
Tissue Sections. Sci. Rep..

[21] Macairan J.-R., Saherwala A., Li F., Monteil-Rivera F., Tufenkji N. (2025). Label-Free Identification and Imaging of Microplastic
and Nanoplastic Biouptake Using Optical Photothermal Infrared Microspectroscopy. Environ. Sci. Technol..

[22] Pięta E., Piergies N., Chrabąszcz K., Banas A., Banaś K., Lo M. K. F., Panek A., Kwiatek W. M., Breese M. B. H., Pogoda K. (2025). Photothermal Infrared Imaging of Nanoplastics in Human
Cells with Nanoscale Resolution. ACS Appl. Mater.
Interfaces.

[23] HassanMazandarani A., Masterson J. M., Querido W., Steplewski A., Zhang Y., Huynh C., Garcia M. M., Fertala A., Pleshko N. (2026). Optical Photothermal Infrared Spectroscopic Assessment
of Microplastics in Tissue Models and Non-Digested Human Tissue Sections. Analyst.

[24] Primpke S., Godejohann M., Gerdts G. (2020). Rapid Identification and Quantification
of Microplastics in the Environment by Quantum Cascade Laser-Based
Hyperspectral Infrared Chemical Imaging. Environ.
Sci. Technol..

[25] Baker M. J., Trevisan J., Bassan P., Bhargava R., Butler H. J., Dorling K. M., Fielden P. R., Fogarty S. W., Fullwood N. J., Heys K. A., Hughes C., Lasch P., Martin-Hirsch P. L., Obinaju B., Sockalingum G. D., Sulé-Suso J., Strong R. J., Walsh M. J., Wood B. R., Gardner P., Martin F. L. (2014). Using Fourier Transform IR Spectroscopy to Analyze
Biological Materials. Nat. Protoc..

[26] Hermes M., Morrish R. B., Huot L., Meng L., Junaid S., Tomko J., Lloyd G. R., Masselink W. T., Tidemand-Lichtenberg P., Pedersen C., Palombo F., Stone N. (2018). Mid-IR Hyperspectral
Imaging for Label-Free Histopathology and Cytology. J. Opt..

[27] Schwaighofer A., Brandstetter M., Lendl B. (2017). Quantum Cascade Lasers (QCLs) in
Biomedical Spectroscopy. Chem. Soc. Rev..

[28] Holub E., Hondl N., Lin K.-L., Parikainen M., Sahlgren C., Lendl B., Ramer G. (2026). Investigating
Spectral
Biomarker Candidates for Migratory Potential in Cancer Cells Using
Micro-FTIR and O-PTIR Spectroscopy. ACS Meas.
Sci. Au.

[29] Xie J., Gowen A., Xu W., Xu J. (2024). Analysing Micro- and
Nanoplastics with Cutting-Edge Infrared Spectroscopy Techniques: A
Critical Review. Anal. Methods.

[30] Liu L., Xu K., Zhang B., Ye Y., Zhang Q., Jiang W. (2021). Cellular Internalization
and Release of Polystyrene Microplastics and Nanoplastics. Sci. Total Environ..

[31] Li Z., Feng C., Wu Y., Guo X. (2020). Impacts of Nanoplastics
on Bivalve: Fluorescence Tracing of Organ Accumulation, Oxidative
Stress and Damage. J. Hazard. Mater..

[32] Chen M., Coleman B., Gaburici L., Prezgot D., Jakubek Z. J., Sivarajah B., Vermaire J. C., Lapen D. R., Velicogna J. R., Princz J. I., Provencher J. F., Zou S. (2024). Identification of Microplastics
Extracted from Field Soils Amended with Municipal Biosolids. Sci. Total Environ..

[33] Prezgot D., Chen M., Leng Y., Gaburici L., Zou S. (2025). Automated
Machine-Learning-Driven Analysis of Microplastics by TGA-FTIR for
Enhanced Identification and Quantification. Anal. Chem..

[34] Zhu Z., Parker W., Wong A. (2023). Leveraging Deep Learning for Automatic
Recognition of Microplastics (MPs) via Focal Plane Array (FPA) Micro-FT-IR
Imaging. Environ. Pollut..

[35] Hufnagl B., Stibi M., Martirosyan H., Wilczek U., Möller J. N., Löder M. G. J., Laforsch C., Lohninger H. (2022). Computer-Assisted
Analysis of Microplastics in Environmental Samples Based on μFTIR
Imaging in Combination with Machine Learning. Environ. Sci. Technol. Lett..

[36] Liu Y., Yao W., Qin F., Zhou L., Zheng Y. (2023). Spectral Classification
of Large-Scale Blended (Micro)­Plastics Using FT-IR Raw Spectra and
Image-Based Machine Learning. Environ. Sci.
Technol..

[37] Coleman B. R. (2025). An Introduction
to Machine Learning Tools for the Analysis of Microplastics in Complex
Matrices. Environ. Sci. Process. Impacts.

[38] Brandt J., Mattsson K., Hassellöv M. (2021). Deep Learning
for Reconstructing
Low-Quality FTIR and Raman Spectra–A Case Study in Microplastic
Analyses. Anal. Chem..

[39] Wang Z., O’ Dwyer K., Muddiman R., Ward T., Camp C. H., Hennelly B. M. (2022). VECTOR: Very Deep Convolutional Autoencoders
for Non-Resonant Background Removal in Broadband Coherent Anti-Stokes
Raman Scattering. J. Raman Spectrosc..

[40] Valensise C. M., Giuseppi A., Vernuccio F., De la Cadena A., Cerullo G., Polli D. (2020). Removing Non-Resonant
Background
from CARS Spectra via Deep Learning. APL Photonics.

[41] Gajjela C. C., Brun M., Mankar R., Corvigno S., Kennedy N., Zhong Y., Liu J., Sood A. K., Mayerich D., Berisha S., Reddy R. (2023). Leveraging Mid-Infrared Spectroscopic
Imaging and Deep Learning for Tissue Subtype Classification in Ovarian
Cancer. Analyst.

[42] Berisha S., Lotfollahi M., Jahanipour J., Gurcan I., Walsh M., Bhargava R., Nguyen H. V., Mayerich D. (2019). Deep Learning for FTIR
Histology: Leveraging Spatial and Spectral Features with Convolutional
Neural Networks. Analyst.

[43] Georgiev D., Fernández-Galian Á., Vilms Pedersen S., Papadopoulos G., Xie R., Stevens M. M., Barahona M. (2024). Hyperspectral
Unmixing for Raman Spectroscopy via Physics-Constrained Autoencoders. Proc. Natl. Acad. Sci. U. S. A..

[44] Xu Y., Zhang L., Du B., Zhang L. (2022). Hyperspectral Anomaly
Detection Based on Machine Learning: An Overview. IEEE J. Sel. Top. Appl. Earth Obs. Remote Sens..

[45] Jiang K., Xie W., Li Y., Lei J., He G., Du Q. (2020). Semisupervised
Spectral Learning with Generative Adversarial Network for Hyperspectral
Anomaly Detection. IEEE Trans. Geosci. Remote
Sens..

[46] Lei J., Fang S., Xie W., Li Y., Chang C.-I. (2020). Discriminative
Reconstruction for Hyperspectral Anomaly Detection with Spectral Learning. IEEE Trans. Geosci. Remote Sens..

[47] Gonzalez C. M., Horrocks T., Wedge D., Holden E. J., Hackman N., Green T. (2023). Anomaly Detection in
Fourier Transform Infrared Spectroscopy of Geological
Specimens Using Variational Autoencoders. Ore
Geol. Rev..

[48] Calin M. A., Parasca S. V. (2022). Automatic Detection
of Basal Cell Carcinoma by Hyperspectral
Imaging. J. Biophotonics.

[49] Jakubek, Z. ; Pegoraro, A. ; Chen, M. ; Maan, Z. ; Zou, S. NPPP-1: Nanoscale Polypropylene Reference Material, 2025. 10.4224/crm.2025.nppp-1.

[50] Pegoraro A. F., Chen M., Sakib S., Jakubek Z. J., Maan Z., Prezgot D., Sallam A., Corriveau D., Vandenberg M., Zou S. (2025). Nanoplastic Reference Materials for
Biological and Methodological Assessment. Microplast.
Nanoplast..

[51] Sakib S., Andoy N. M. O., Yang J. Y. C., Galang A., Sullan R. M. A., Zou S. (2025). Antimicrobial and Anti-Inflammatory
Effects of Polyethyleneimine-Modified
Polydopamine Nanoparticles on a Burn-Injured Skin Model. Biomater. Sci..

[52] Erb, D. Pybaselines: A Python Library of Algorithms for the Baseline Correction of Experimental Data. 10.5281/zenodo.5608581.

[53] Han, Q. ; Peng, S. ; Xie, Q. ; Wu, Y. ; Zhang, G. Iterative Reweighted Quantile Regression Using Augmented Lagrangian Optimization for Baseline Correction. In 2018 5th International Conference on Information Science and Control Engineering (ICISCE), 2018; pp 280–284. 10.1109/ICISCE.2018.00066.

[54] Ruff L., Kauffmann J. R., Vandermeulen R. A., Montavon G., Samek W., Kloft M., Dietterich T. G., Müller K.-R. (2021). A Unifying
Review of Deep and Shallow Anomaly Detection. Proc. IEEE.

[55] Zhu, D. ; Du, B. ; Zhang, L. EDLAD: An Encoder-Decoder Long Short-Term Memory Network-Based Anomaly Detector for Hyperspectral Images. In 2021 IEEE International Geoscience and Remote Sensing Symposium IGARSS, 2021; pp 4412–4415. 10.1109/IGARSS47720.2021.9553145.

[56] Han M., Dang Y., Han J., Han M., Dang Y., Han J. (2024). Denoising and Baseline Correction
Methods for Raman Spectroscopy
Based on Convolutional Autoencoder: A Unified Solution. Sensors.

[57] Zhang Z.-M., Chen S., Liang Y.-Z. (2010). Baseline
Correction Using Adaptive
Iteratively Reweighted Penalized Least Squares. Analyst.

[58] Bhattacharya P., Lin S., Turner J. P., Ke P. C. (2010). Physical Adsorption of Charged Plastic
Nanoparticles Affects Algal Photosynthesis. J. Phys. Chem. C.

[59] Thomas N. W., Jenkins P. G., Howard K. A., Smith M. W., Lavelle E. C., Holland J., Davis S. S. (1996). Particle Uptake and Translocation
across Epithelial Membranes. J. Anat..

[60] Han W., Cui J., Sun G., Miao X., Pufang Z., Nannan L. (2024). Nano-Sized
Microplastics Exposure Induces Skin Cell Senescence via Triggering
the Mitochondrial Localization of GSDMD. Environ.
Pollut..

[61] Zhang X., Qiu J., Zhao J., Li X., Liu L. (2020). Complex Refractive
Indices Measurements of Polymers in Infrared Bands. J. Quant. Spectrosc. Radiat. Transfer.

