# Supplementary material for: Autoencoder-Based Detection of Nanoplastics in Biological Matrices via Infrared Hyperspectral Imaging
Source: ACS Meas Sci Au. 2026 Jul 18;6(4):1129–40. doi: 10.1021/acsmeasuresciau.6c00067 (PMC13495456; doi:10.1021/acsmeasuresciau.6c00067)
Supplement: Supplementary file 1 [file tg6c00067_si_001.pdf]

Supporting information for

Autoencoder-Based Detection of Nanoplastics in Biological Matrices via Infrared Hyperspectral Imaging

Daniel Prezgot,[1] Sadman Sakib,[1] Maohui Chen,[1] Adrian F. Pegoraro,[1] David Corriveau,[1] and Shan Zou[1]**

[1] Nanoscale Measurement, Metrology Research Centre, National Research Council Canada, 100 Sussex Drive, Ottawa, Ontario, K1A 0R6, Canada

*Corresponding authors:

Daniel Prezgot – Metrology Research Center, National Research Council Canada, Ottawa, Ontario, Canada; orcid.org/0000-0001-6498-4422; Email: daniel.prezgot@nrc-cnrc.gc.ca

Shan Zou, Metrology Research Center, National Research Council Canada, Ottawa, Ontario, Canada; orcid.org/0000-0002-2480-6821; Email: shan.zou@nrc-cnrc.gc.ca

TABLE OF CONTENTS

Item	Description	Page
**Table S1**	Hyperparameters investigated during model tuning	**S2**
**Figure S1**	Example of full preprocessing -> Image process of the guided anomaly detection framework	**S3**
**Table S2**	Reconstruction error & RMS noise in residual spectra for model reconstructions of a test dataset	**S4**
**Figure S2**	Comparison of pre-processing (baseline correction) methods to reconstructed spectra and model performance	**S4**
**Figure S3**	Comparison of heatmaps generated and model performance on images processed with a generalized vs a specialized model	**S5**
**Figure S4**	Comparison of reconstructed spectra produced by models with different sizes of training data.	**S6**
**Table S3**	Metrics for comparison of models produced with different sizes of training data	**S6**
**Figure S5**	Examples at very high, and low exposure levels, contrasting guided and unguided detection approaches.	**S7**
**Figure S6**	Alternative LOD-calibration curve, in a narrower range above the LOD	**S8**

Table S1 Hyperparameter tuning summary for autoencoder models presented in this work: fully-connected (FC), convolutional neural network (CNN), and hybrid CNN-FC. The final hyperparameter values and associated model score are presented.

Model	Hyperparameter	Value Range	Final Value(s) [f]	Model Score [g]
FC	encoder depth [a]	2 – 4	2	5.74
	layer size	8 – 256	265, 32, 256	
	dropout [b]	0 – 0.2	0	
	γ [c]	0 – 1×10^4	1×10^2	
	learning rate [d]	1×10^{-3} – 1×10^{-5}	1×10^{-4}	
CNN	encoder depth [a]	2 – 4	3	5.02
	channels [e]	4 – 1024	16, 64, 256	
	kernels [e]	3 – 19	7, 5, 5, 5, 5, 7	
	strides [e]	2 – 4	3, 3, 3, 3, 3, 3	
	dropout [b]	0 – 0.2	0	
	γ [c]	0 – 1×10^4	1×10^2	
	learning rate [d]	1×10^{-3} – 1×10^{-5}	1×10^{-4}	
CNN-FC	encoder depth [a]	2 – 4	3	7.20
	channels [e]	4 – 256	16, 64, 256	
	kernels [e]	3 – 15	7, 5, 5	
	strides [e]	2 – 4	3, 3, 3	
	decoder depth	1 – 2	1	
	layer size	32 – 1024	1024	
	dropout [b]	0 – 0.2	0	
	γ [c]	0 – 1×10^4	1×10^2	
	learning rate [d]	1×10^{-3} – 1×10^{-5}	1×10^{-4}	

[a] – Defined as the number of hidden layers between the input and bottleneck layers, including the bottleneck but not the input. FC & CNN architectures had symmetric decoders, giving (2n – 1) total hidden layers.

[b] – Dropout layers were added to the final layer of the encoder before the bottleneck. Since 0 dropout performed best, layers were not included in diagrams (Figure 1, main text)

[c] – γ is the balancing factor for the raw MSE & derivative MSE in the loss function (equation 1, main text)

[d] – learning rate and number of epochs were also evaluated by looking at the learning curve for a smooth gradient with little noise during training. 5 epochs was chosen as a value which universally reached the learning curve floor.

[e] – channels, kernels, and strides were chosen in combinations such that the input and output dimensions (460) were equal, occasionally using an output padding of 1 in the last layer to satisfy this condition.

[f] – Final hyperparameters for models, which are those that maximized Model Score.

[g] – Maximum score, where models were scored based on their ability to minimize total reconstruction error (RE, equation 2, main text), while maximizing errors at 1376 cm^{-1} & 1456 cm^{-1} (strongest characteristic bands for PP) in a validation sample containing NPPP-1. The score was calculated as:

$$Model\ Score = \frac{RE_{(1376)} + RE_{(1456)}}{RE_{total}}$$

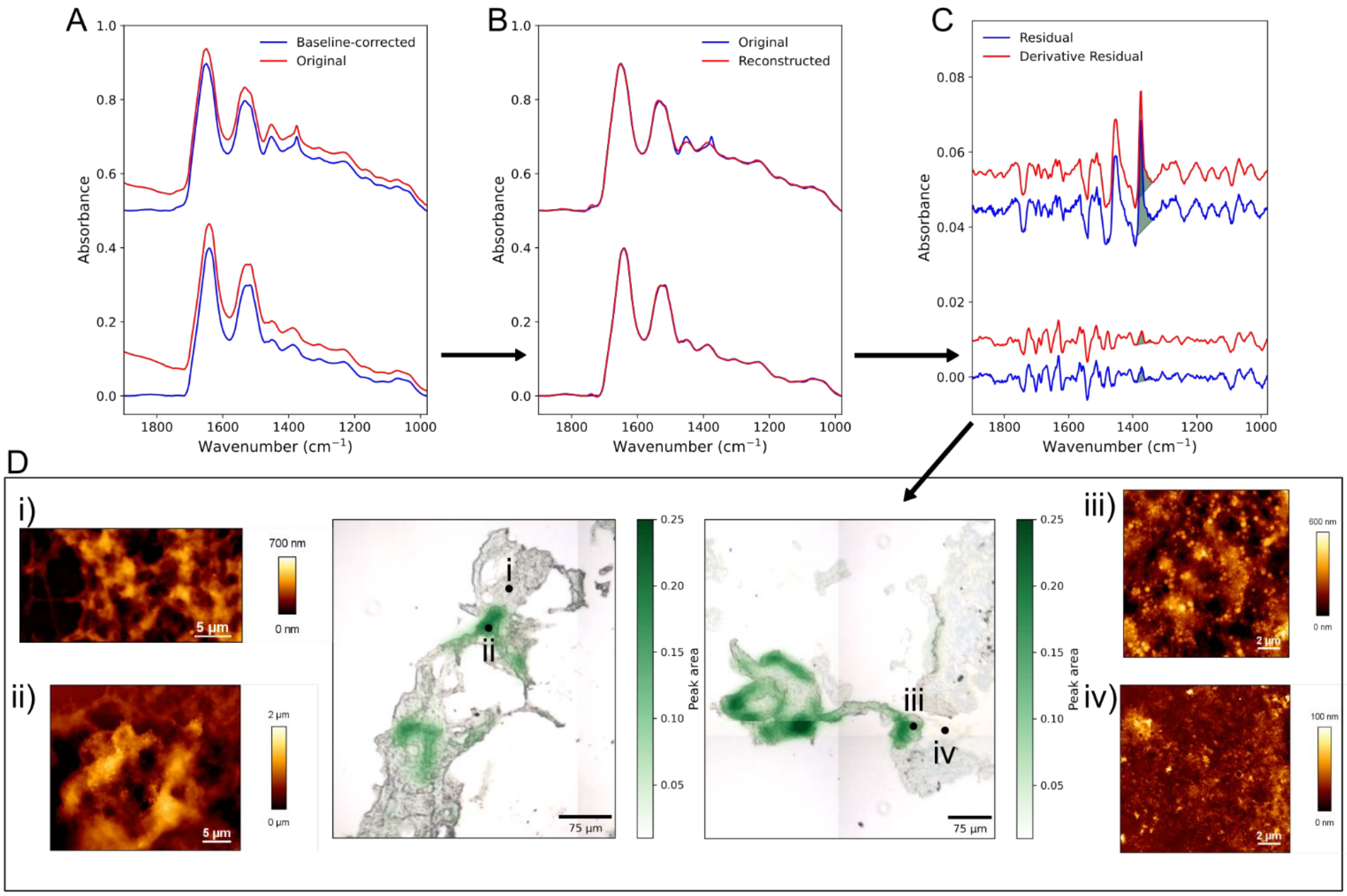

Figure S1 Demonstration of the full workflow of the guided anomaly detection process, from raw spectral data to the produced heatmap, demonstrated on a NPPP-1 area and an "empty" area of a sample hyperspectral image. A) Rubberband baseline correction preserves the overall spectral shape – merely drops the "floor" of the spectrum in lieu of normalization. B) Original and reconstructed spectra produced from the CNN-FC model. The non-plastics containing reconstruction overlaps the original near-perfectly. C) Residual spectra extracted as either the difference or the integrated difference in residuals. The latter approach reduces high frequency noise in the reconstruction. D) By integrating the 1378 cm^{-1} signal (highlighted in C), a heatmap of NPPP-1 content is produced. AFM images confirm the presence of NPs where predicted in the heatmap (ii, iii), and the absence of NPs in the biological matrix (i) and background substrate (iv).

Table S2 – Reconstruction error and average RMS noise of the residual spectra of a test image of an HSIO not exposed to NPs. The RMS noise was evaluated only in areas of the image above a threshold IR absorption from the organoid, ignoring the background of the image.

Model	Reconstruction Error	RMS noise
FC	0.2566	0.00224
CNN	0.1393	0.00075
CNN-FC	0.1549	0.00108

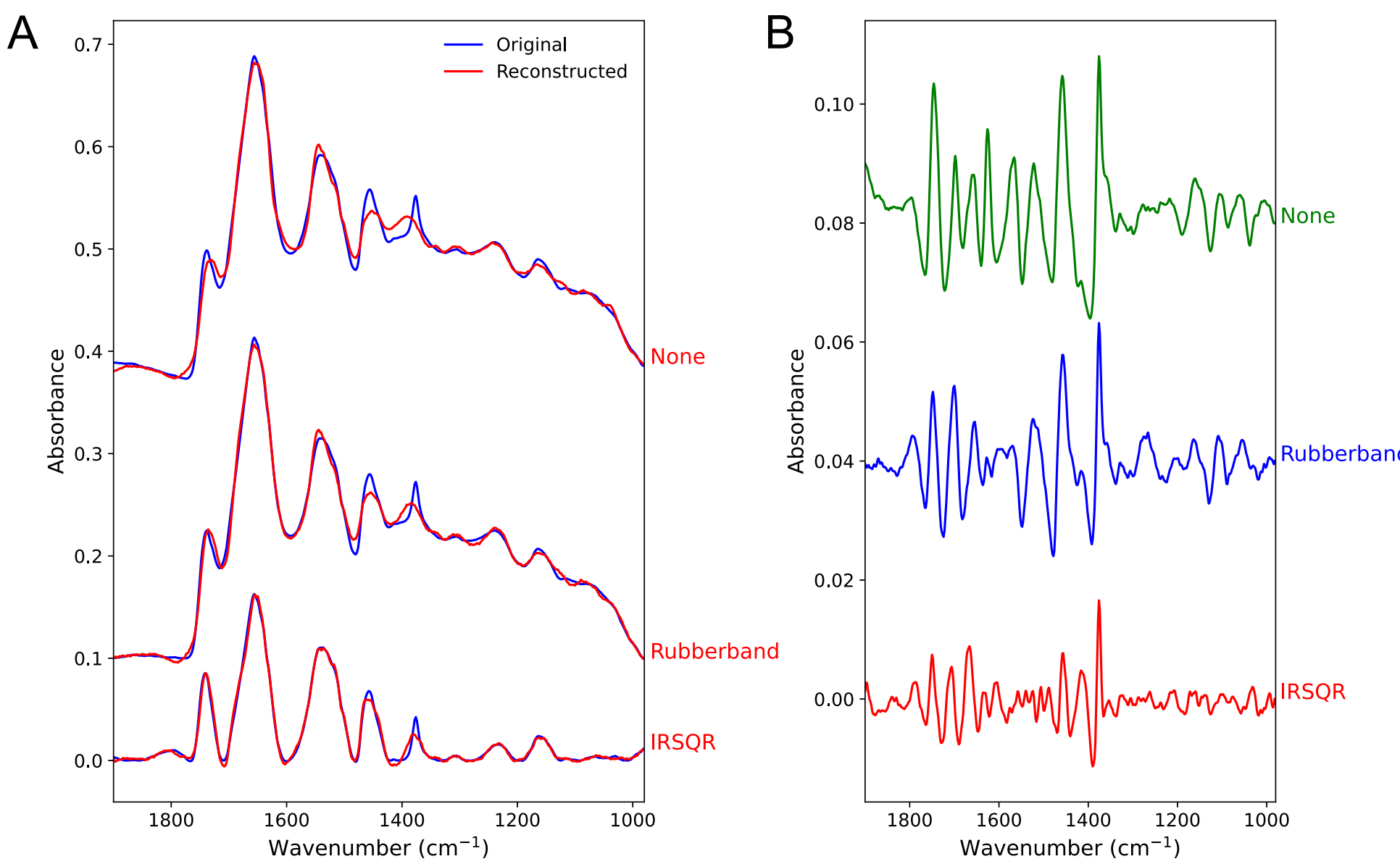

Figure S2 A) An example of original and reconstructed data using the CNN-FC model trained and evaluated with different baseline correction techniques. B) The resulting residual spectrum for each method. With no baseline correction, the signal-to-noise (measured as peak height from the base of the 1378 cm^{-1}) was 3.47, which improved to 4.06 with rubberband baseline correction. IRSQR correction further reduced noise and increased the SNR to 6.68, but spectral characteristics, such as the peak width and relative height of the 1456 cm-1 mode, were not preserved.

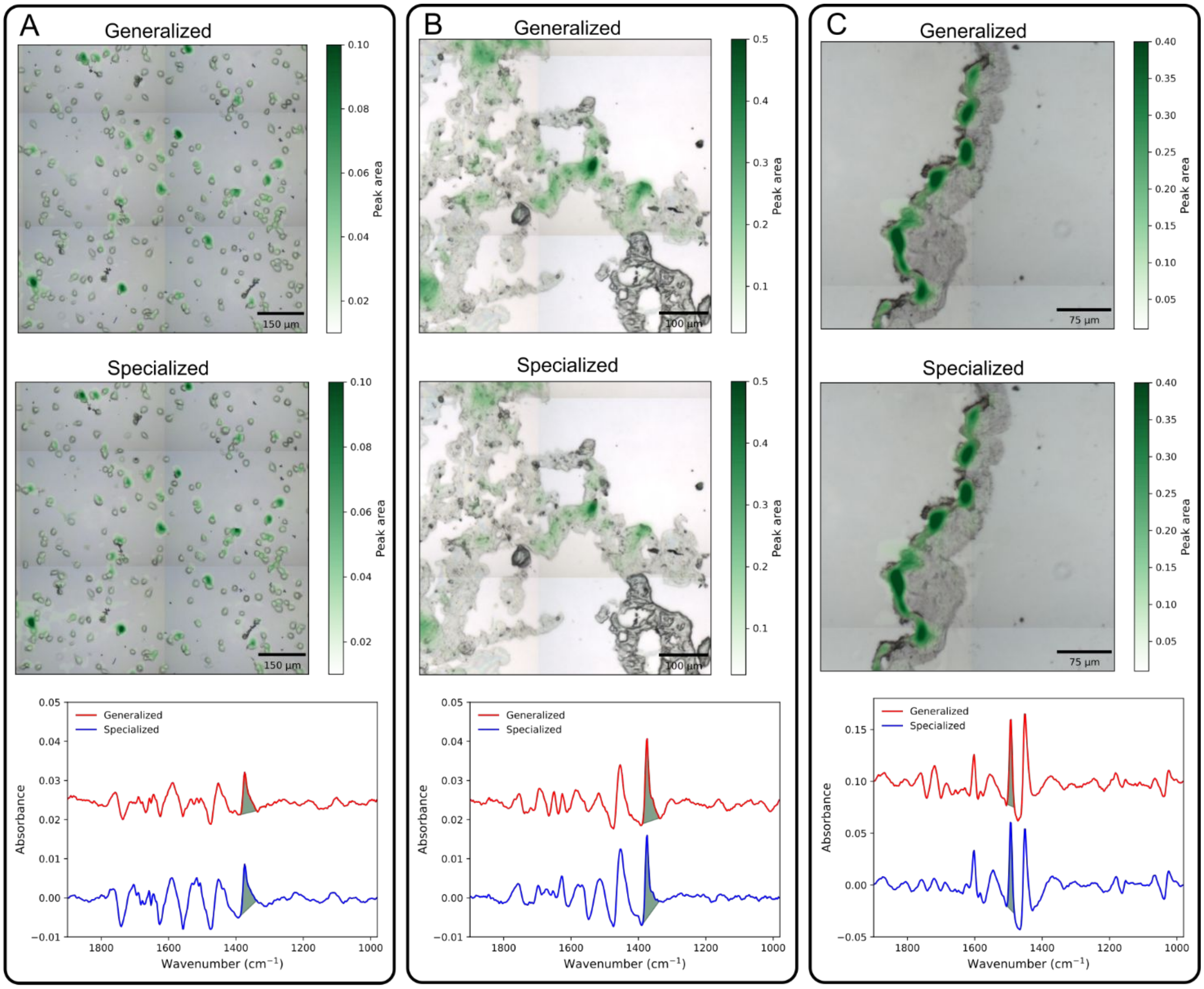

Figure S3 Nanoscale plastic content heatmap and example residual spectra produced from CNN-FC models trained using a generalized or specialized training dataset. The generalized set contained data from HIEC (A), HSIO (B) and HSM(C) sources, whereas the specialized set contained only system-specific data for each source. Both datasets were assembled to contain roughly similar numbers of spectra (1.2–1.5 M). Differences in images are subtle and nearly indistinguishable, while the overall residual spectral quality was comparable. In general, system-specific models showed reduced overall reconstruction error, but similar, and in some cases slightly reduced, signal-to-noise in regions containing nanoscale plastic accumulations.

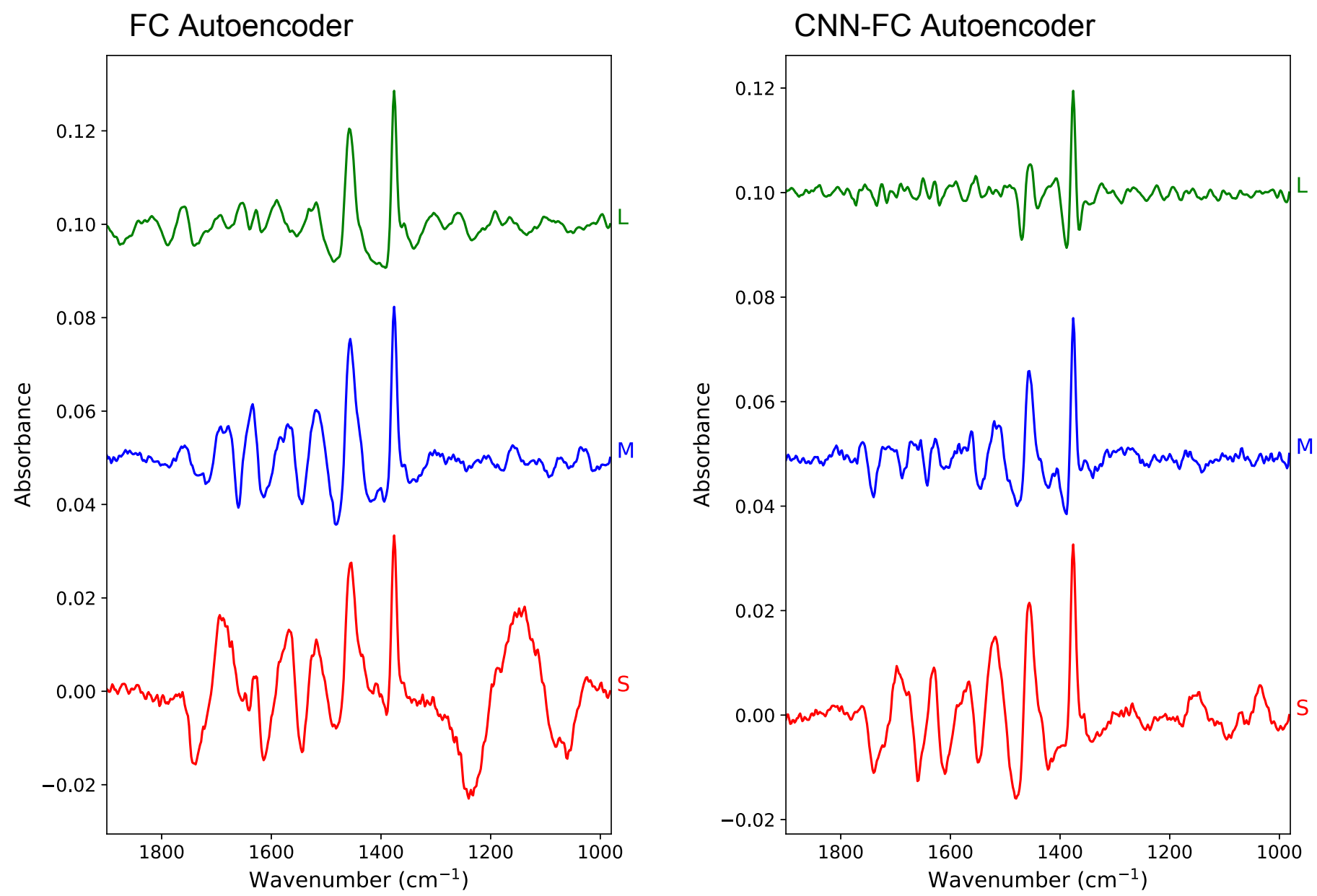

Figure S4 Sample residual spectra produced from FC and CNN-FC models trained on small (S), medium (M), and large (L) datasets. The datasets consisted of 0.28 M, 1.36 M, and 8.24 M spectra for the S, M, and L datasets, respectively. In both datasets, the S training set produced large reconstruction errors and poor SNR. Reconstruction quality and SNR increased with the M dataset for both models. Using a larger dataset improved SNR but had a negligible effect on the FC model's overall quality. In the CNN-FC model, the larger dataset was detrimental, leading to overgeneralization in spectral reconstruction and the loss of characteristic spectral features. Full metrics are reported in Table S3.

Table S3 Metrics associated with Figure S4. SNR was calculated for the sample spectra in Table S4, as the total peak height @ 1378 cm^{-1} / RMS noise in regions of the spectra excluding 1300–1500 cm^{-1}. Reconstruction error and model score were evaluated using a validation image containing NPPP-1. It should be noted that all models were trained for 5 epochs; the total number of spectra used for training was not reflected in the reported size of the training set.

**Model**	**FC**			**CNN-FC**		
**Training set**	**SNR (spectrum)**	**Reconstruction Error (image)**	**Model Score**	**SNR (spectrum)**	**Reconstruction Error (image)**	**Model Score**
**S**	3.82	0.896	2.89	4.00	0.435	4.07
**M**	4.14	0.244	5.54	5.56	0.156	6.46
**L**	5.21	0.314	5.54	8.22	0.110	4.70

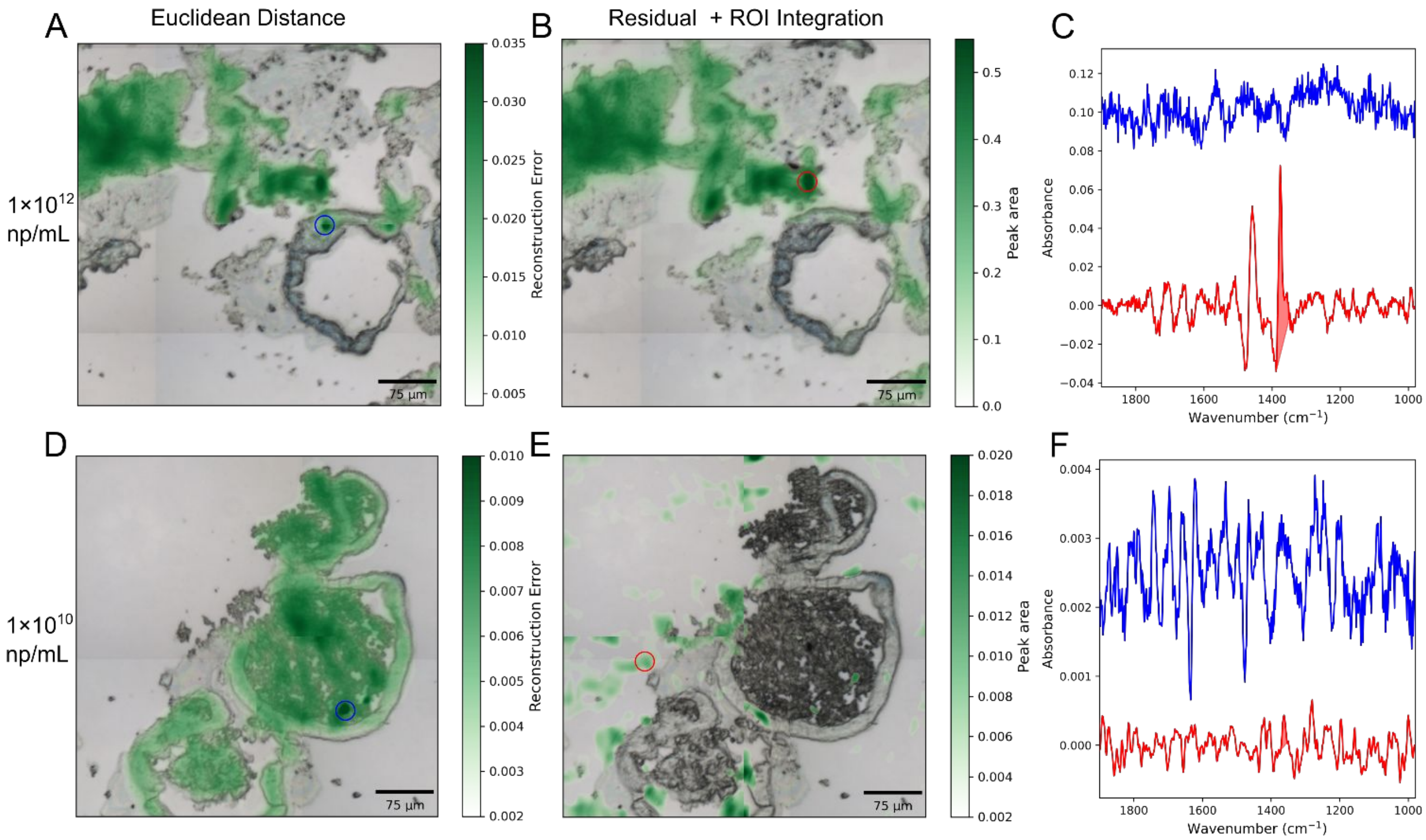

Figure S5 Example heatmaps and spectra from HSIOs exposed to NPPP-1 at concentrations of 10^{12} particles/mL (A,B,C) and 10^{10} particles/mL (D,E,F). Heatmaps A and D were acquired with an unguided Euclidean distance metric, whereas B and E were retrieved by integrating the residual around ~ 1378 cm^{-1}. At 10^{12} particles/mL exposure, the heatmaps (A, B) are largely similar; heatmap (A) highlights a false positive arising from reconstruction errors (C, blue trace). Heatmaps at 10^{10} particles/mL (D & E) differ significantly, highlighting distinct false positives. The guided approach highlights reconstruction errors in strongly absorbing regions of the organoid, whereas heatmap E highlights background noise, which happens to show a peak-like structure within the ROI.

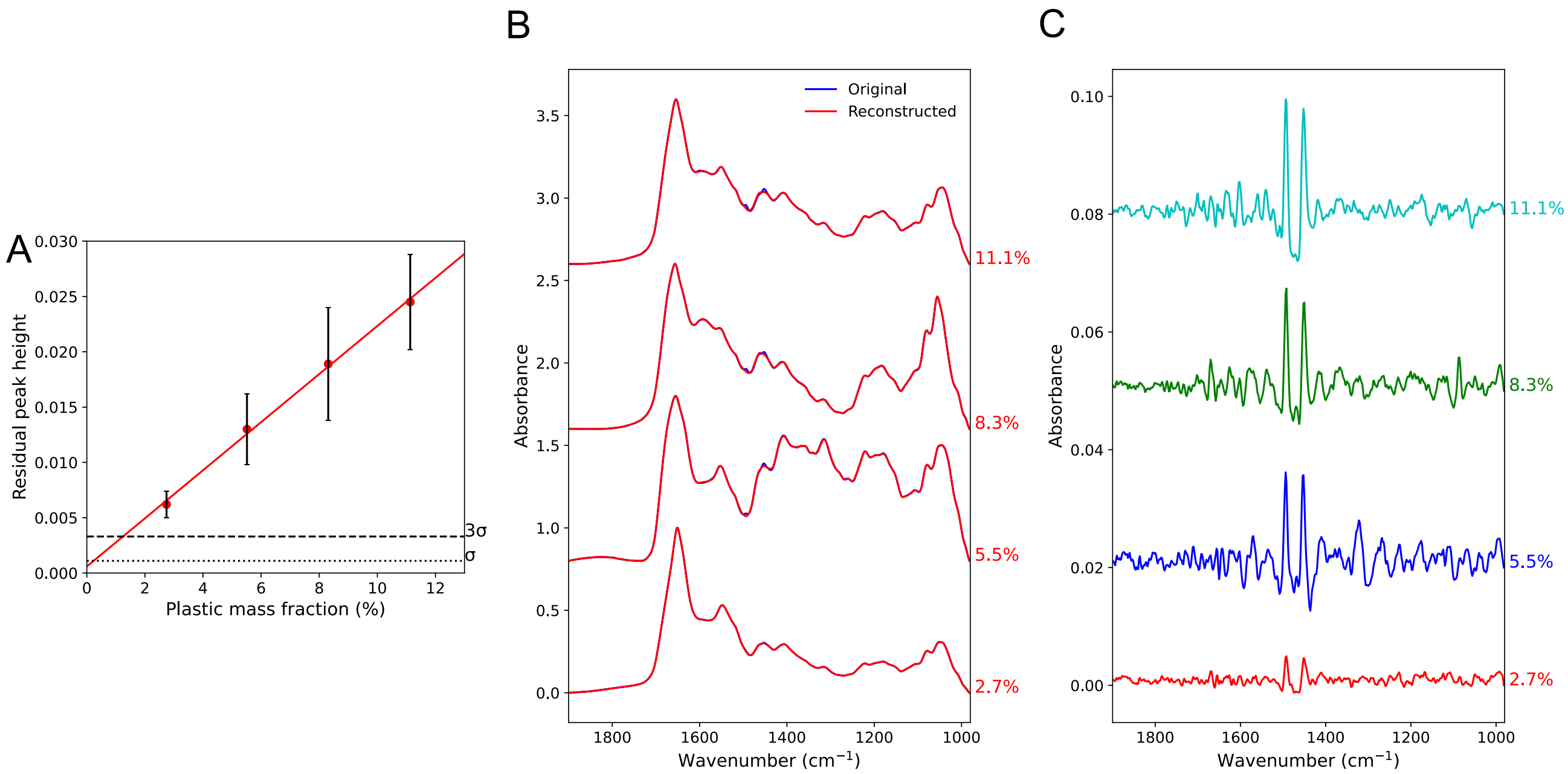

Figure S6 A) Calibration curve of 50 nm polystyrene spheres relative to total mass of extracellular matrix (ECM), performed on a narrower set of mass fractions. B) Associated example original and reconstructed spectra from the samples used to create the calibration curve. C) associated example residual spectra. These ECM samples were less spatially consistent than those shown in Figure 6, resulting in higher overall measurement error and lower SNR. The projected 3σ LOD increased slightly (0.7% -> 1.2%) relative to Figure 6, remaining consistent with the previous observation but highlighting the importance of model fitting and residual noise level to the detection limit.
